# Engineered calcium-regulated affinity protein for efficient internalization and lysosomal toxin delivery

**DOI:** 10.1073/pnas.2509081122

**Published:** 2025-11-25

**Authors:** Malin Jönsson, Marit Möller, Leon Schierholz, Nicolai Dorka, Hanna Tegel, Emma Lundberg, Mathias Uhlén, Magnus Wolf-Watz, Hjalmar Brismar, Sophia Hober

**Affiliations:** ^a^Department of Protein Science, SciLifeLab, KTH-Royal Institute of Technology, Stockholm, Sweden; ^b^Department of Molecular Biology, Umeå University, Umeå SE-901 87, Sweden; ^c^Department of Bioengineering, Stanford University, Stanford, CA 4525; ^d^Department of Chemistry, Umeå University, Umeå SE-901 87, Sweden; ^e^Department of Applied Physics, SciLifeLab, KTH-Royal Institute of Technology, Solna 171 65, Sweden

**Keywords:** calcium-regulated affinity, conditional targeting, drug-conjugate, endosomal release, cancer

## Abstract

The emerging strategy of protein–drug conjugates (PDCs) for targeted cancer therapy holds great potential to improve treatment efficacy by specifically targeting cancer biomarkers and delivering toxic payloads directly to tumor cells, minimizing off-target toxicity. The success of this approach depends on the internalization and retention of the payload in target cells. This study introduces a method using a small protein domain engineered for conditional target affinity, enabling lysosomal trafficking independent of the biological fate of the receptor. Specifically, we describe the development of an EGF receptor binder, CaRA_EGFR_, with calcium-regulated affinity (CaRA), meaning the target binding strength is tailored by the available calcium concentration. This allows for endosomal dissociation, as calcium levels are lower in endosomes than in the bloodstream. Affinity measurements and structural modeling reveal the molecular basis of the calcium modulated affinity. Live cell imaging demonstrates efficient internalization and lysosomal trafficking of the calcium-dependent domain, while the EGF receptor is recycled to the membrane. When used as a drug carrier, CaRA_EGFR_ effectively delivers the toxin to the lysosomes, resulting in potent cytotoxicity with an IC50 of 0.8 nM in EGFR-expressing cancer cells

## Significance

This study introduces a calcium-regulated protein domain (CaRA_EGFR_) engineered for efficient internalization and targeted toxin delivery in cancer cells. By exploiting calcium gradients to control binding affinity, this approach enables precise, receptor-independent drug delivery to lysosomes, achieving potent cytotoxicity (IC50 = 0.8 nM) with the potential to avoid receptor downregulation. This proof-of-concept marks the use of calcium-regulated affinity (CaRA) in a small protein scaffold, offering a groundbreaking strategy to enhance specificity, efficacy, and safety in targeted cancer therapy.

Overexpression of receptors promoting cell proliferation is a hallmark of cancer and a viable strategy for selective cancer therapy is to target these receptors, yet safety concerns of toxicity in healthy tissues have been raised (1–3). Developed drug conjugates consist of a target-seeking moiety, such as an antibody or an alternative scaffold protein, designed to deliver a cytotoxic payload specifically to cancer cells while minimizing damage to healthy tissues (4, 5). However, the efficacy of current therapies may be limited due to an inherent basal receptor expression, or treatment resistance resulting from a receptor downregulation, or mutation in the targeted cancer receptor (6, 7). Moreover, most cytotoxic payloads require intracellular delivery, which makes strong binding to the receptor potentially detrimental to the therapeutic effect. To address this challenge, a strategy involving cleavable linkers between the target-seeking moiety and the carried payload has been developed. These linkers are designed to preferentially release the payload shortly after the compound enters the cell. The cleavage of these linkers is primarily facilitated by chemical or enzymatic processes, which are highly sensitive to the concentrations of other species in the local cellular environment (8).

Engineering strategies for conditional target protein interaction are emerging as a promising approach to circumvent the challenges of toxicity in healthy tissues (9–11). Conditional target affinity can be achieved through allosteric regulation (12–14), by addition of a regulatory binding motif into a tumor-targeting antibody, or by making use of histidine protonation. As a result, the target interaction becomes dependent on known characteristics of the tumor microenvironment such as elevated levels of ATP or acidity. (12–14) Incorporation of a tunable target affinity enabling a more directed therapeutic activation has been shown to increase the therapeutic window and improve drug efficacy (10–11, 15). The advantages of conditional activation are particularly relevant for drug-conjugates, as treatment efficiency is often limited by dose-dependent systemic toxicity. Additionally, a reduction in the surface expression of target antigen has been previously observed, making conditional activation a promising strategy to enhance efficacy while minimizing adverse effects (16–18). Apart from protein engineering strategies for specific conditional activation in the tumor microenvironment, an innovative design strategy for enhanced intracellular release has demonstrated promising results in tumor-bearing mouse xenografts (19). This strategy is focused on a more efficient payload delivery by incorporating pH dependency, which enables endosomal receptor dissociation. In this study, the pH dependency was incorporated into a clinically approved antibody–drug conjugate rendering its affinity for human epidermal growth factor receptor 2 (HER2) higher at physiological pH compared to intracellular acidic pH, resulting in increased lysosomal payload delivery and improved therapeutic efficiency over the acid-insensitive original antibody–drug conjugate. This was believed to be an effect of enabling endosomal receptor dissociation in the lower pH of intracellular compartments in comparison to the neutral pH in blood circulation, leading to lysosomal payload entry and unbound receptor recycling (19). Independent or combined, these conditional activation strategies provide opportunities for future fine-tuned tumor-selective therapies.

The epidermal growth factor receptor (EGFR) is overexpressed in a variety of solid tumors such as lung and colorectal cancer (20). Upon internalization, EGFR has been reported to undergo different trafficking routes depending on the ligand that binds the receptor and the specific HER-family member with which it has heterodimerized (21–25). Hence, once EGFR is internalized and enters the endosomes, it can either be recycled back to the cell surface or transported to the lysosomes for degradation (26–30).

**Fig. 1.**
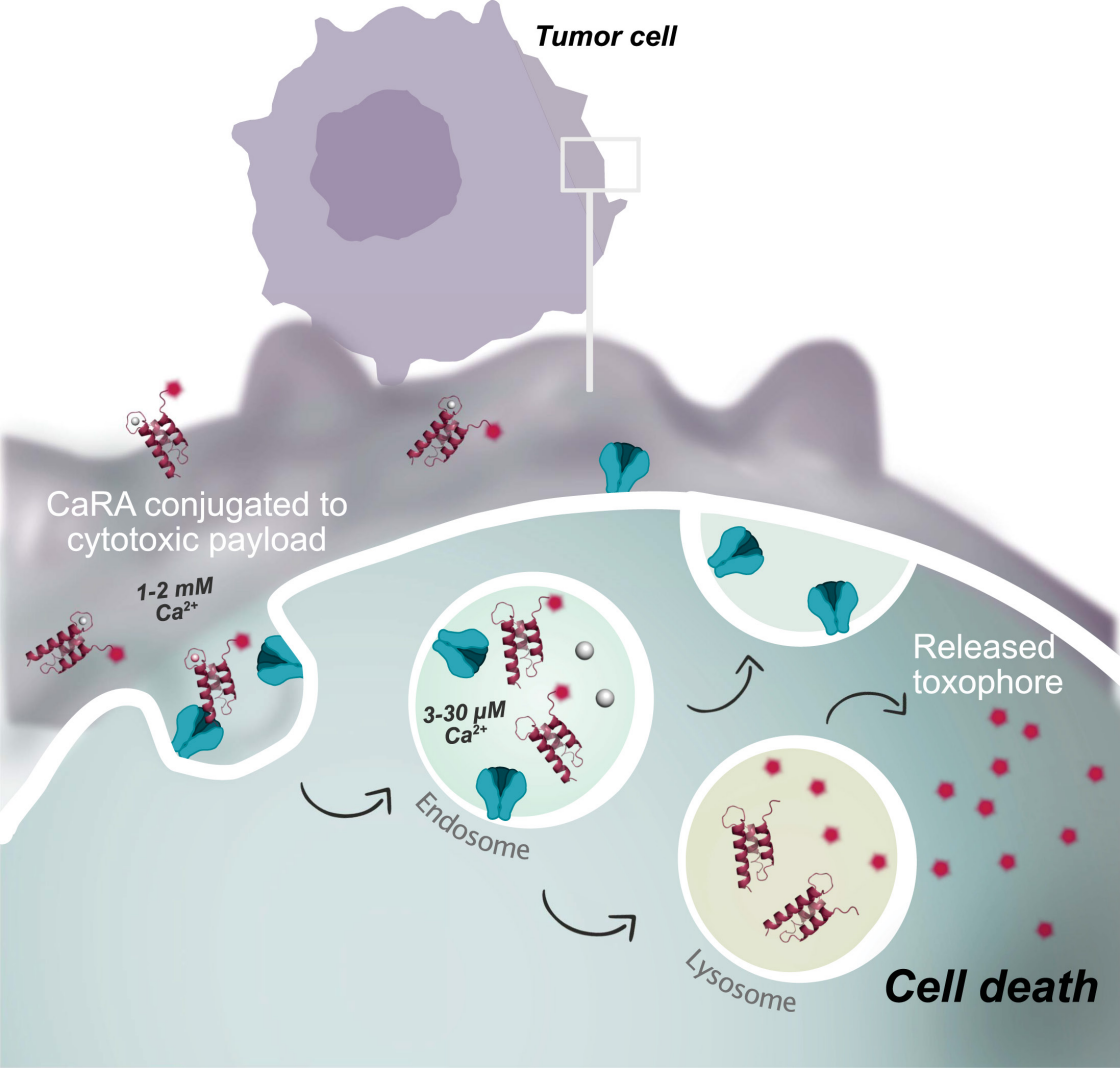
Concept of a calcium-regulated affinity (CaRA) binder enabling endosomal receptor dissociation, for recycling of unbound receptor, aimed to enhance intracellular delivery of a cytotoxic payload to cancer cells. Due to the difference in calcium concentration between extracellular and intracellular compartments, a CaRA binder interacts with its target with high affinity in the higher calcium concentration while a target dissociation would be enabled at the lower calcium concentration found in the endosome. This would allow for recycling of unbound receptor and might lead to a constant surface expression of the receptor, offering continuous efficient delivery of the next CaRA molecule. Noteworthy, the calcium mechanism enables the fate of a CaRA molecule to be decoupled from the fate of the targeted receptor, so that CaRA remains within the cells and, when conjugated to a cytotoxic payload, a tumor-killing effect upon entry into the lysosomes can be achieved.

We have recently reported on a combinatorial protein library, denoted CaRA, for selection of calcium-regulated affinity (CaRA) binders (31). The alternative scaffold protein within the library has an inherent calcium-binding motif and is histidine-enriched to allow for selection of calcium dependency as well as pH dependency. We hypothesized that using this library for selection against overexpressed cancer receptors like EGFR could generate fine-tuned binders enabling new treatment regimes. Envisioned are conditional high-affinity binders designed to exploit the change in calcium concentration between endosomes (μM-range) and circulation (mM range) (32, 33). By conjugating the CaRA binders to a toxic payload, specific delivery to the lysosomes could be achieved and thereby the cancer could be effectively eradicated, independent of the trafficking fate of the cancer receptor itself (Fig. 1). Here, we present the selection, engineering, and characterization of CaRA_EGFR_ which is shown to have a conditional target affinity for EGFR-expressing cancer cells. Biolayer interferometry studies clearly demonstrate that the target affinity of these binders can be tailored by the available calcium levels. Noteworthy, live cell confocal microscopy data illustrate the internalization and lysosomal delivery of CaRA_EGFR_ and in vitro cytotoxicity studies show a resulting IC50 of 0.8 nM when CaRA_EGFR_ is conjugated to a toxin. Taken together, these findings underscore the promising therapeutic potential of CaRA binders.

## Results

### Generation of a CaRA Binder Conditionally Targeting EGFR.

A strategy for efficient delivery into cellular compartments is to generate conditional binders able to interact with higher affinity to their target in the extracellular environment than in the intracellular environment, enabling recycling of unbound target receptor and directed dissociation of the binder upon internalization into cells. There is about a 15-fold change in proton concentration, pH, when going from plasma into endosomes while there is a minimum of 150-fold difference in calcium concentration (32–34). To take advantage of both differences, a library for development of calcium-regulated affinity binders (CaRA) designed to have a high prevalence of histidine residues was used to generate a conditionally targeting alternative scaffold protein toward EGFR (31). Phage display selections were performed toward a recombinantly produced monomeric version of the extracellular domain of EGFR (*SI Appendix*, Fig. S1) with a strategy aimed to promote association to the target at pH 7.4 and a calcium concentration of 1 mM, and dissociation in a calcium-free environment with acidic pH to mimic the in vivo conditions of the endosome. Deep sequencing data of the selection output combined with a high-throughput kinetic screening procedure (35) resulted in a candidate referred to as CaRA_EGFR_ (*SI Appendix*, Fig. S1).

### Switch-Like Binding Between CaRA_EGFR_ and EGFR in Response to Calcium Concentration.

**Fig. 2.**
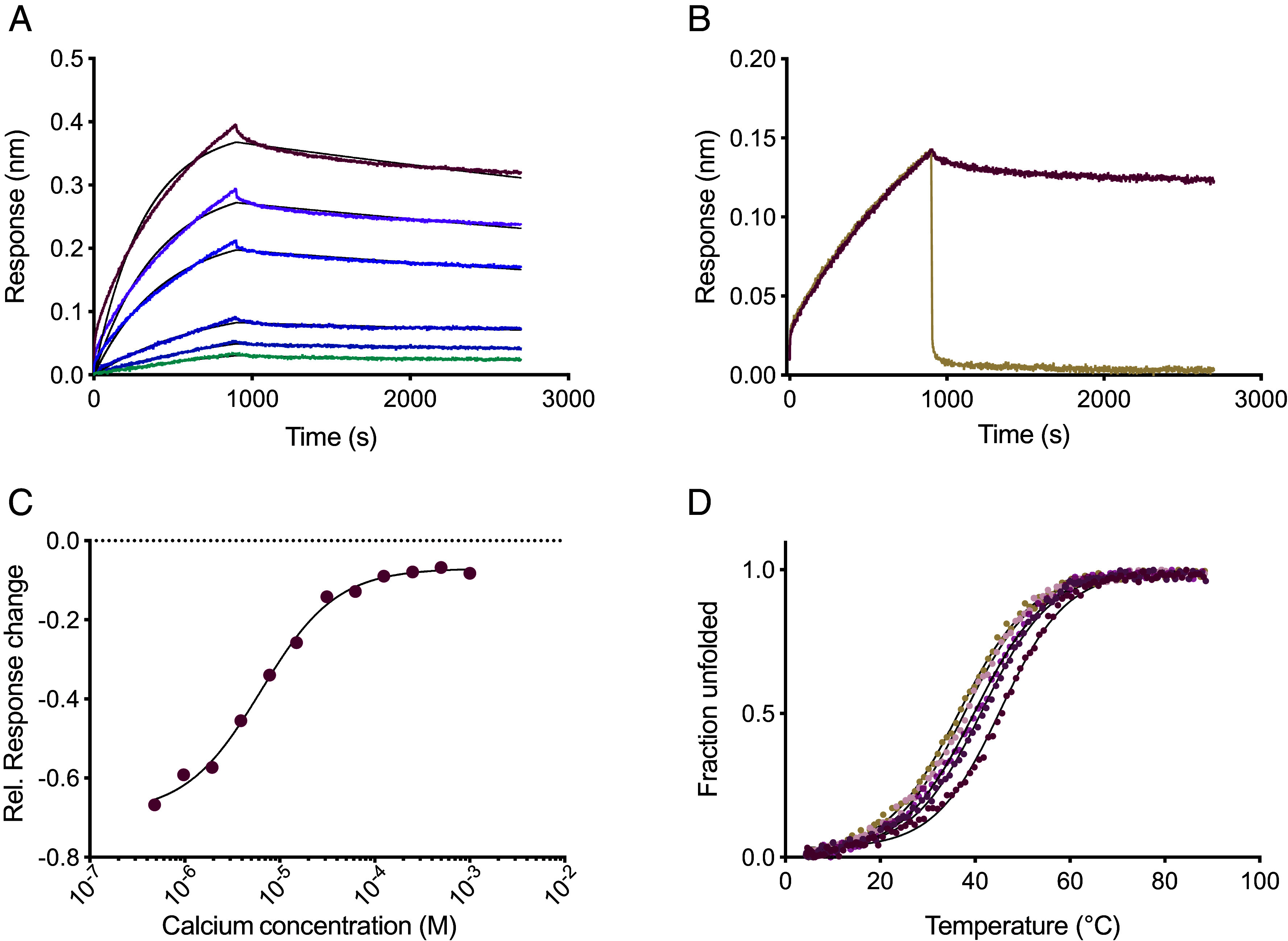
Calcium-dependent binding analysis of CaRA_EGFR_ with the recombinant EGFR. (*A*) Biolayer interferometry was used to assess the binding to the target EGFR in solution (twofold dilution series from 2,000 nM to 63 nM) by capturing CaRA_EGFR_-ABD on HSA-immobilized sensors. Binding kinetics was evaluated in buffer supplemented with 1 mM calcium at pH 7.4. Kinetic fits to a 1:1 binding model are shown in black. Averages of the association rate constant k_a_, the dissociation rate constant k_d_, and the dissociation equilibrium constant K_D_ resulted in k_a_ = 5.8 * 10^3^ M^−1^ s^−1^, k_d_ = 1.0 * 10^−4^ s^−1^ and a K_D_ of 20 nM. (*B*) Evaluation of calcium-dependent release of the target EGFR (at 500 nM) in 1 mM calcium and dissociation in either 1 mM calcium (burgundy) or 1 μM calcium (beige). (*C*) The calcium affinity was estimated by conducting the same experiment as in Fig. 2*B* with associating to EGFR in 1 mM calcium and dissociating in different calcium concentrations ranging from 1 mM to 0.48 μM. The response change after 30 s dissociation was normalized against the maximum binding response for the individual curves at the different release conditions. The data were fitted to a sigmoidal curve, from which the calcium affinity can be estimated to be around 6.4 μM. (*D*) Thermal stability of CaRA_EGFR_ was quantified with CD spectroscopy at 221 nm by exposing the protein to a temperature gradient from 4 °C to 95 °C. The experiment was performed in a buffer supplemented with various calcium concentrations (1 mM, 500 μM, 250 μM, 50 μM, and 1 μM) at pH 7.4. The melting temperature increased by 10 °C following an increase in calcium concentration from 1 μM (beige) to 1 mM (burgundy), highlighting the secondary structure’s dependence on calcium.

The calcium-regulated binding behavior of CaRA_EGFR_ to the extracellular domain of the EGF receptor was analyzed using biolayer interferometry. Capturing of the CaRA molecule on the sensors enabled investigation of target interaction in various calcium environments. Under physiological conditions, with pH 7.4 and a 1 mM calcium concentration, the interaction have a slow on-rate (k_a_ = 5.8 * 10^3^ M^−1^ s^−1^), but also a slow off-rate (k_d_ = 1.0 * 10^−4^ s^−1^), resulting in a moderate to high-affinity equilibrium constant (K_D_) of around 20 nM (Fig. 2*A*). The K_D_ was also determined by surface plasmon resonance (*SI Appendix*, Fig. S2), yielding a comparable affinity of 5.5 nM. To investigate the calcium-dependent release from the target, the complex was associated under the same conditions while dissociation was observed at a lower calcium concentration without a change in pH. Following a 1,000-fold lower calcium concentration, the CaRA_EGFR_ molecule releases its target rapidly and reaches baseline levels within 30 seconds (Fig. 2*B*), indicating that the molecule is able to bind EGFR in a switch-like manner, solely dependent on the calcium concentration of the surrounding environment and independent of pH. The fast release is demonstrated also at an endosomal pH of 5.0 when combined with an endosomal calcium concentration (*SI Appendix*, Fig. S3). This reflects physiologically relevant conditions during endocytic sorting, where both calcium levels and pH are simultaneously reduced. Moreover, observing the dissociation at different calcium concentrations indicated that the release was finely tunable, especially highlighting the very fast off-rate seen at a concentration of 1 μM calcium which would be mimicking the endosomal environment. Interestingly, titration of the target binding strength at different calcium concentrations also provides an estimate of the calcium affinity which is calculated to around 6 μM (Fig. 2*C*).

These findings prompted us to further investigate whether the functional change seen could be related to the structure and thermal stability of the CaRA molecule at the different calcium concentrations. CaRA_EGFR_ retains binding functionality and structural integrity under the conditions where receptor-binding is envisioned, in the high calcium concentration observed in circulation, whereas the binding is weakened in a low calcium environment mimicking the endosome. Thus, a stability investigation covering the pH and calcium concentrations known to be relevant for endocytosis was performed. The construct was diluted in buffers containing a twofold dilution series from 1 mM calcium, mimicking the circulation, to 1 μM calcium, to simulate endosomal compartments, and a pH varying from 7.4 to 6.0. Circular dichroism (CD) spectroscopy measured at 221 nm was used to quantify the melting temperature (T_m_) of the construct for each of these conditions (Fig. 2*D* and *SI Appendix*, Fig. S4). The experiments demonstrate that the T_m_ does not vary much within the pH interval tested while it increases with about 10 °C when the calcium concentration is increased from 1 μM to 1 mM, resulting in a maximal T_m_ of around 46 °C with 1 mM calcium and a minimal T_m_ of 36 °C obtained in the μM-calcium concentration environment (*SI Appendix*, Table S1). The increase in T_m_ following increased calcium concentration shows that CaRA_EGFR_ harbors an inherent affinity for calcium that is independent of binding to EGFR.

### Flow Cytometric Analysis Shows Specific Interaction of CaRA_EGFR_ with Human EGFR-Expressing Cells.

The selective and specific interaction of the CaRA binder with the endogenous receptor was assessed using human cancer cell lines with various expression levels of EGFR; including A-431 cells (high expression), MDA-MB-468, SK-BR3, SK-OV-3, NCI-H292, and BxPC-3 cells (intermediate expression), MCF-7 and Ramos cells (low or no detected EGFR-expression) (36, 37). With the exception of lymphoma (Ramos), these cell lines also express other HER-family member receptors, namely HER2 and HER3 to various degrees (*SI Appendix*, Table S2).

**Fig. 3.**
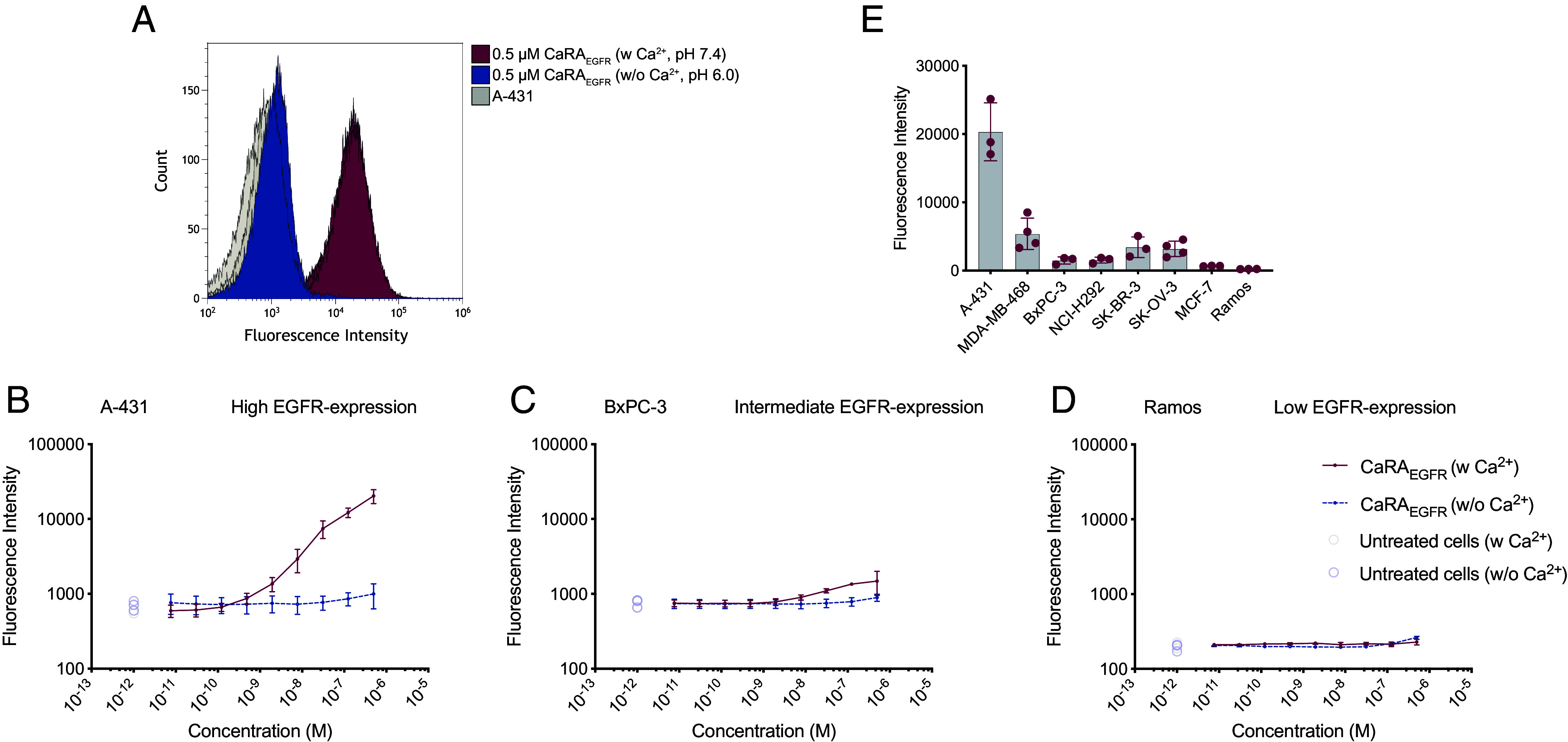
Flow cytometric binding analysis of the calcium-dependent interaction between CaRA_EGFR_ and various cancer cell lines. The conditional binding of CaRA_EGFR_ was evaluated on cancer cell lines with high (A-431), intermediate (BxPC-3), and low (Ramos) EGFR-expression. (*A*) For the high EGFR-expressing A-431 cells, a shift in binding signal could be observed for the triplicates of 500 nM CaRA_EGFR_ in the presence of calcium (burgundy) but not in the calcium-free experiment (blue) where the latter is overlapping with the control samples consisting of untreated cells (gray) which have been incubated in the respective buffers either with or without calcium. From the measured median fluorescent intensity of averaged biologically replicate samples with calculated variance shown as error bars, an on-cell affinity curve was graphed for a concentration series of CaRA_EGFR_ ranging from 7.62 pM to 500 nM toward the cancer-derived cell lines with varying EGFR-expression; (*B*) high (A-431), (*C*) intermediate (BxPC-3), or (*D*) low (Ramos). The estimated affinity to the endogenous EGF receptor resulted in around 7 nM. (*E*) The measured median fluorescent intensity for the highest CaRA_EGFR_ concentration tested (500 nM) was plotted for the various cell lines and clearly shows that the obtained signals vary with the described EGFR-expression (*SI Appendix*, Table S2), indicating a specific target interaction.

For the flow cytometry analysis, the cells were incubated with a fourfold dilution series of site-specifically labeled CaRA_EGFR_-Alexa488 ranging from 7.62 pM to 500 nM and a resulting concentration-dependent shift was observed in the fluorescence signal (Fig. 3 and *SI Appendix*, Fig. S5) in the presence of calcium. When the equivalent concentration gradient of CaRA_EGFR_ was run in the absence of calcium, no binding could be detected (Fig. 3 *A*–*D* and *SI Appendix*, Fig. S5). An affinity for the endogenous receptor of approximately 7 nM was estimated based on the steepest slope of the resulting sigmoidal curve, which was generated by plotting the median fluorescence intensity of each biological replicate against the applied concentration of CaRA_EGFR_ (Fig. 3 *B*–*D* and *SI Appendix*, Fig. S5). The obtained median fluorescent intensity for the highest CaRA_EGFR_ concentration of 500 nM with the averaged signals from a blank cell sample subtracted was plotted for each cell line (Fig. 3*E*). It can be clearly seen that no significant binding was detected when CaRA_EGFR_ was allowed to interact with the negative or low expressing cell lines (Ramos and MCF-7) while increasing signals were obtained with increasing EGF receptor expression, independent of the HER2 and HER3 expression of these cells (*SI Appendix*, Table S2). Hence, CaRA_EGFR_ is shown to interact specifically and selectively with cells expressing endogenous EGFR which is a promising indication for minimal off-target effects.

### Experimentally Validated AlphaFold 3 Model of CaRA_EGFR_:EGFR.

**Fig. 4.**
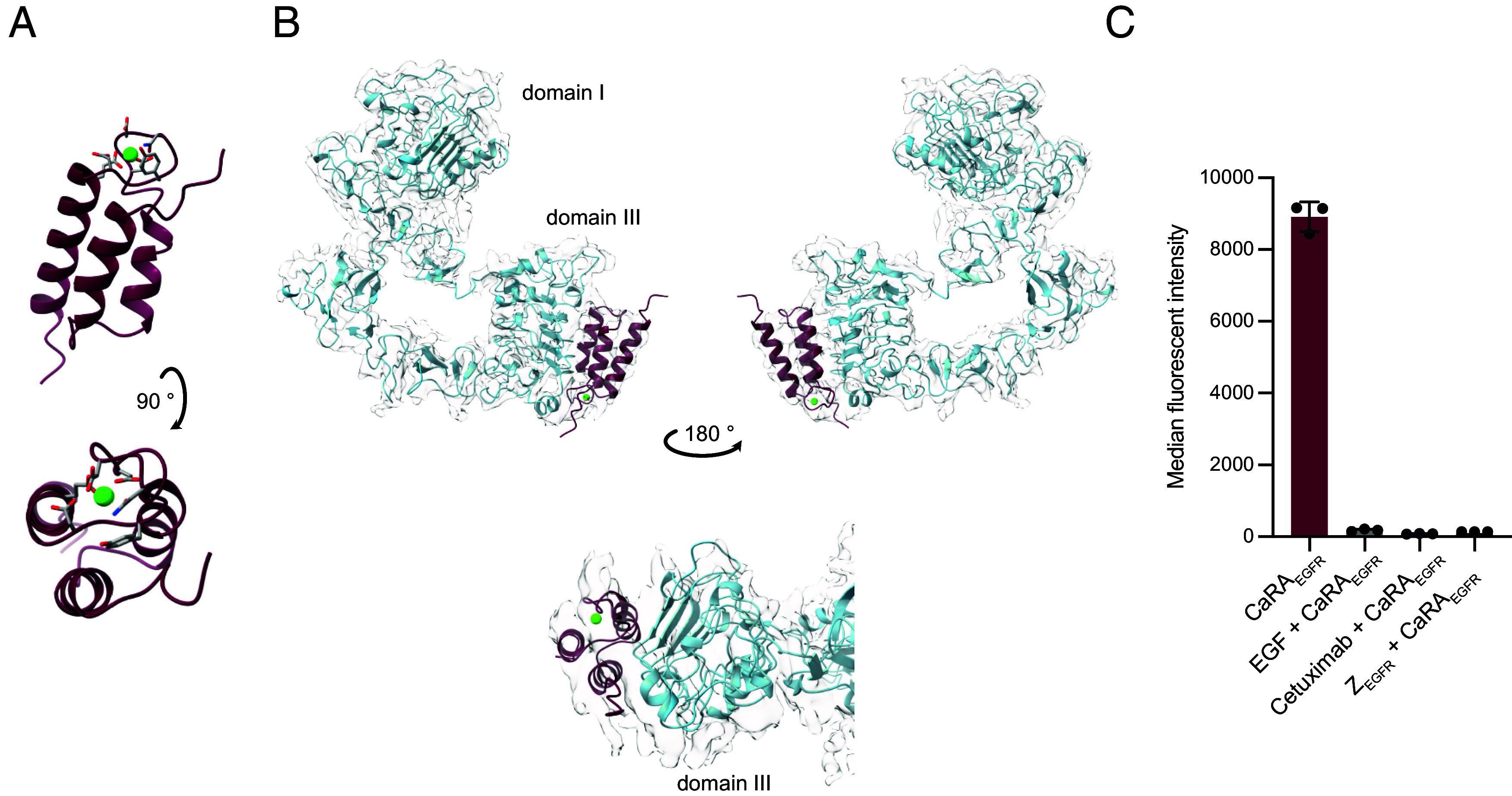
Structural modeling and validation of the CaRA_EGFR_;EGFR complex. (*A*) AlphaFold 3 (38) prediction of CaRA_EGFR_ with a Ca^2+^ ion as a ligand (green) is presented. The predicted structure is shown from both the *Front* and *Top* views (90 ° rotated), with the *Top* view highlighting the amino acid side chains predicted to contribute to calcium binding. (*B*) Best fit of the CaRA_EGFR_-EGFR complex model to a moderate resolution cryo-EM density map electron cloud (~6 Å) and a closer view of the AlphaFold 3 predicted target–binder interaction on domain III of EGFR below. CaRA_EFGR_ is shown in burgundy, and the extracellular domain of EGFR is shown in blue. (*C*) Epitope binning experiments showing specific blocking of CaRA_EGFR_ (100 nM) with equimolar ratios of the ligand EGF, the monoclonal antibody Cetuximab, or the affibody Z_EGFR:2377_, in flow cytometry, supporting the predicted binding position in (*B*). The error bars show the SD as calculated from triplicate samples.

To further characterize the CaRA_EGFR_ binder and the interaction with its target EGFR, we modeled the complex with the structure prediction tool AlphaFold 3 (38). The model was subsequently validated with single particle cryo-EM at intermediate resolution, binding surface analysis and functional data. The AlphaFold 3 prediction of CaRA_EGFR_:EGFR suggests that the calcium ion is coordinated in the loop between helix 2 and 3, as expected, with primary contributions from Asp, Asn, and Glu residues (Fig. 4*A*). The RMSD of the CaRA_EGFR_ model relative to the crystallographic structure of parental Z_Ca_ in complex with Ca^2+^ (chain E in 6FGO.pdb) is 0.7 Å, which establishes their strong structural similarity (*SI Appendix*, Fig. S6*A*) (31). In the modeled complex structure, helices 2 and 3 of CaRA_EGFR_ interact with a binding site located in domain III of the extracellular domain of EGFR (Fig. 4*B*). A single particle cryo-EM map at ~6.0 Å resolution contains a density that clearly accounts for the binder based on model fitting and docking simulations. This fit validates the predicted structure of the complex including the positioning of CaRA_EGFR_.

In the selection process to generate the EGFR binder, 18 amino acid residues were mutated relative to the parental protein (31). A display of these residues on the modeled complex (*SI Appendix*, Fig. S6*B*) shows that most of the changed residues are in direct contact with EGFR, which serves as an additional validation of the modeled complex. Further, an epitope mapping experiment on EGFR-expressing cells preincubated with either the natural ligand epidermal growth factor (EGF), the antibody Cetuximab, or the affibody (Z_EGFR:2377_) shows specific blocking of CaRA_EGFR_ (Fig. 4*C*), also suggesting that all molecules share a similar epitope on domain III (39) (*SI Appendix*, Fig. S7), as demonstrated previously for Z_EGFR:2377_ in relation to EGF and Cetuximab (36, 40) Taken together, we present an Alphafold3 model of the CaRA_EGFR_:EGFR complex that was extensively validated with independent experiments.

### Confocal Micrographs Show the Decoupled Biological Fate of CaRA_EGFR_ and Its Targeted Receptor.

To evaluate the biological fate of a conditional high-affinity binding domain upon target interaction, confocal microscopy was used to visualize the internalization of CaRA_EGFR_ and the cell surface exposed EGF receptor expressed by HEK293T cells transfected with EGFR and A-431 cancer cells expressing endogenous EGFR. CaRA_EGFR_ which has been site-specifically C-terminally labeled with Alexa488 was added to HEK293T cells transfected with a plasmid encoding EGFR with Fusion Red in the C-terminus (EGFR-FR), which allowed for recording of the binding kinetics on live cells (*SI Appendix*, Fig. S8 *A* and *B*). CaRA_EGFR_ displayed a fast and specific interaction with the cell surface exposed EGFR upon addition to the cells, characterized by reaching receptor saturation within 10 seconds after addition (*SI Appendix*, Fig. S8*B*). Although colocalization of CaRA_EGFR_ and EGFR-FR can be clearly seen at images captured after 16 s and 400 s, the signals are separated again in the 24 h micrographs (*SI Appendix*, Fig. S8*C*), indicating a receptor-independent retention of CaRA_EGFR_ within the cells.

**Fig. 5.**
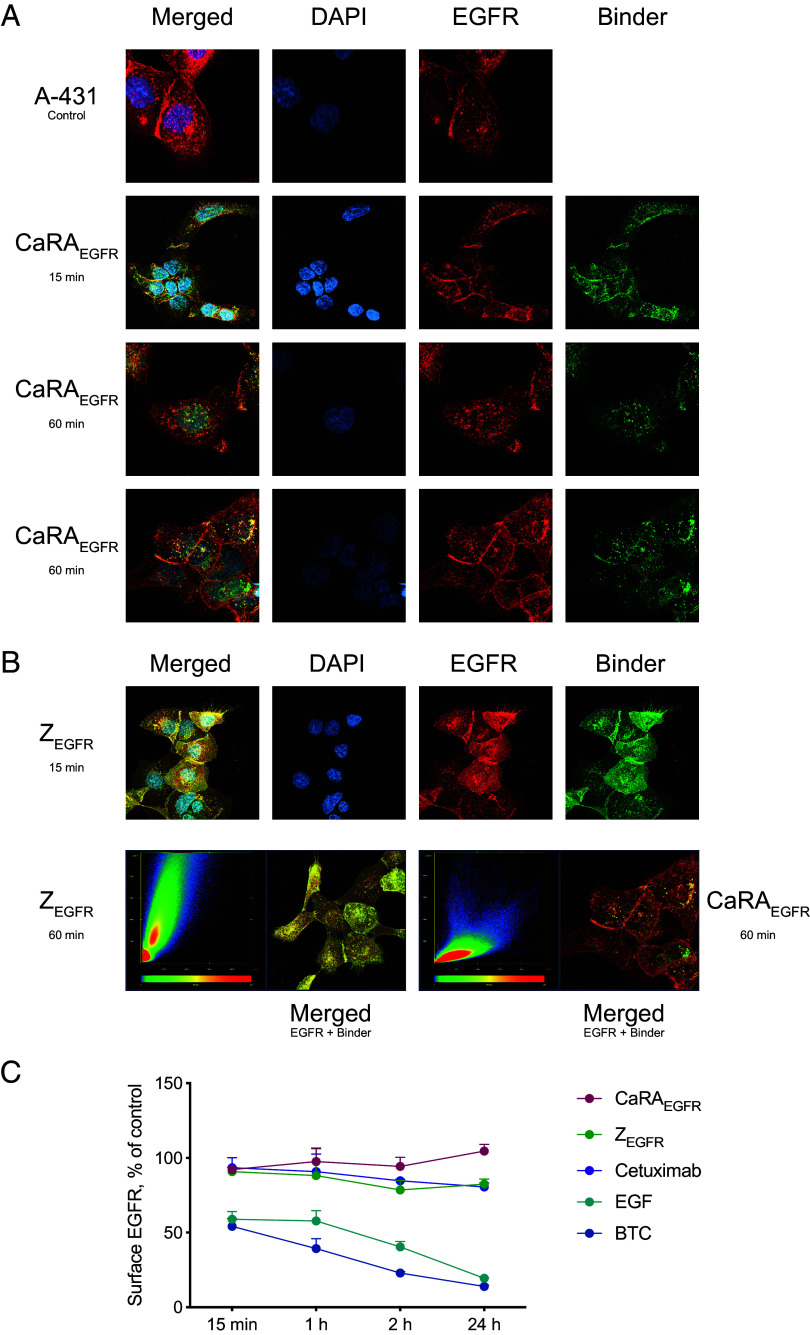
Cellular localization of CaRA_EGFR_ or Z_EGFR:2377_ and the targeted EGF receptor and EGFR expression levels after incubation with binders. (*A* and *B*) A decoupled biological fate is shown for CaRA_EGFR_ and EGFR. For CaRA_EGFR_, a high intracellular retention is shown compared to the nonconditionally binding Z_EGFR:2377_ control. The Alexa488 fluorescently labeled CaRA_EGFR_ or Z_EGFR:2377_ (green) was added to EGFR-expressing A-431 cells and incubated at 37 °C for 15 min or 60 min prior to fixation of the cells and subsequent labeling of EGFR (red, Alexa647) and the cell nuclei (blue, DAPI). In the 15 min time frame, CaRA_EGFR_-Alexa488 shows colocalization with the Alexa647-labeled EGFR on the membrane as does the nonconditionally interacting Z_EGFR:2377_. Internalized CaRA_EGFR_ is already detectable after 15 min of incubation but becomes more pronounced in the 60 min samples, where numerous cells display distinct green vesicles near the nuclei. These vesicles are clearly separated from the red-labeled EGFR, which remains predominantly at the cell surface. In both the 15 min and 60 min time frame, Z_EGFR:2377_-Alexa488 shows colocalization with the Alexa647-labeled EGFR on the membrane. When visualizing the colocalization of Alexa647-labeled EGFR and Alexa488-labeled Z_EGFR:2377_, the signals largely overlap, indicating strong colocalization between the high-affinity binder and its target receptor even after 60 min. In contrast, internalized CaRA_EGFR_ is clearly visible after 60 min and appears as a distinct population from the labeled EGFR, shown in red in the colocalization graph. In the 2D histograms the red EGFR signal is shown on the x-axis and green CaRA_EGFR_ signal on the y-axis is shown. The colocalization plot shows that Z_EGFR:2377_ colocalizes with after 60 min, also shown in yellow in the cell image. However, a discrete localization of binder without EGFR is shown for CaRA_EGFR_ after 60 min. This is also visible as solid green vesicle-like structures in the cell image. (*C*) Quantitative flow cytometry of NCI-H292 cells incubated for 15 min to 24 h with EGF, BTC, CaRA_EGFR_, Z_EGFR:2377_, or Cetuximab shows that CaRA_EGFR_ does not downregulate EGFR surface expression within the tested time frame, whereas clear downregulation is observed for the natural ligands, Z_EGFR:2377_, and Cetuximab. Data are shown as mean out of triplicates.

To further investigate the apparently distinct intracellular fates of CaRA_EGFR_ and its target, the EGF receptor, A-431 cells were incubated for 15 min or 60 min at 37 °C with either the conditionally binding CaRA_EGFR_ molecule or a high-affinity EGFR-binding Affibody molecule (Z_EGFR:2377_) sharing a similar epitope as CaRA_EGFR_ (36, 40). Both constructs were site-specifically labeled at the C-terminus with Alexa488. After the specified incubation times, cells were fixed and subsequently stained for EGFR. In Fig. 5, it can be clearly seen that after 15 min CaRA_EGFR_ mainly colocalizes with EGFR at the cell membrane. However, green vesicles containing CaRA_EGFR_ but lacking Alexa647-labeled EGFR begin to emerge. Following 60 min of incubation, these CaRA_EGFR_-positive vesicles become more prominent, while EGFR remains primarily localized at the cell membrane (Fig. 5). Further, for the nonconditionally EGFR-binding Affibody molecule, an overlapping localization with EGFR is seen both after 15 min and after 60 min incubation. Interestingly, when comparing the colocalization with EGFR between the nonconditionally binding Z_EGFR:2377_ and the conditionally interacting CaRA_EGFR_ it can be demonstrated that the fate of a nonconditional binding molecule seems more closely linked to the biological fate of the targeted receptor (Fig. 5). Moreover, quantitative flow cytometry data where NCI-H292 cells are incubated for 15 min to 24 h with either the natural ligands of the EGF receptor (EGF or BTC) (24), CaRA_EGFR_, Z_EGFR:2377_, or Cetuximab confirms that CaRA_EGFR_ does not seem to downregulate the EGFR cell surface expression during the investigated time frame, whereas the downregulation is very clear for the natural ligands and also seems to occur for the non-calcium-dependent Z_EGFR:2377_ and Cetuximab (Fig. 5*C*).

### Fluorescence Microscopy Reveals that CaRA_EGFR_ Is Trafficked to the Lysosomes.

**Fig. 6.**
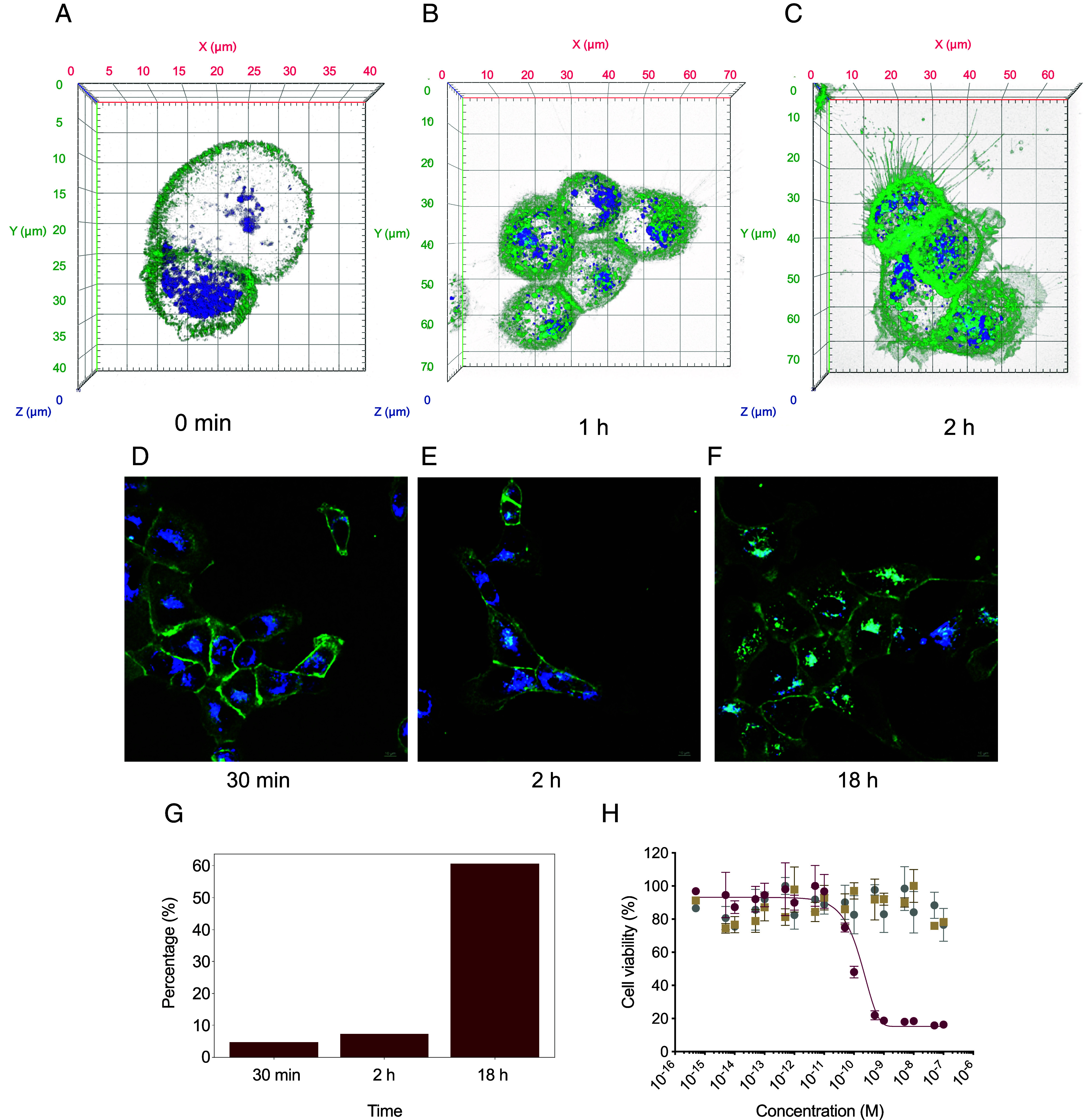
Confocal microscopy images of A-431 cells expressing EGFR exposed to CaRAEGFR show lysosomal accumulation and in vitro cytotoxicity assay results in an IC50 of 0.8 nM. (A-F) Alexa488 fluorescently labeled CaRA_EGFR_ is shown in green, lysosome maker in blue, and colocalization appears as turquoise. (*A*–*C*) A-431 cells incubated at 37 °C with Alexa488 fluorescently labeled CaRA_EGFR_ for 0 min, 1 h, and 2 h. (*D*–*F*) A-431 cells incubated at room temperature with Alexa488 fluorescently labeled CaRA_EGFR_ for 10 min before washout and increased temperature to 37 °C to initiate internalization of the EGFR. Confocal micrographs from the center of the cells were captured after 30 min (*D*), 2 h (*E*), and 18 h (*F*). CaRA_EGFR_ is initially localized at the membrane, where EGFR is commonly expressed. The images show colocalization of signals of CaRA_EGFR_ and the lysosome dye as early as after 30 min, with a following time-dependent increase (*G*). (*H*) The cytotoxicity of CaRA_EGFR_ conjugated to the toxin DM1 was determined by incubating serial dilutions of the conjugate or controls with the cell line A-431 as indicated. Ztaq-DM1 was used as a nontargeting control conjugated to DM1 and CaRA_EGFR_ is the targeting molecule without the cytotoxin. The cell viability was measured after 48 h. The viability of untreated cells in complete medium was used for normalization to 100% viability. The data points represent the average of triplicates in the same assay with error bars showing the SD. The IC50 of 0.8 ± 0.3 nM was calculated from three independent experiments.

To investigate the intracellular destination of CaRA_EGFR_ following internalization, A-431 cells were labeled with a lysosome-specific dye and exposed to site-specifically labeled CaRA_EGFR_-Alexa488, alongside a control consisting of a nonconditional EGFR-binding Affibody molecule (36, 40). Confocal microscopy was used to monitor the cells in two independent experiments, one of which included an extended incubation period. In the first experiment, cells were maintained at 37 °C and monitored continuously for 2 h following the addition of the fluorescently labeled constructs (Fig. 6 *A*–*C* and *SI Appendix*, Fig. S9). In the second experiment, confocal images were captured at discrete time points, 30 min, 2 h, and 18 h postincubation, to assess temporal changes in intracellular localization. Both experiments show initial binding of CaRA_EGFR_ to the cell membrane where EGFR is commonly located, followed by colocalization with the lysosome dye after incubation at 37 °C for more than 30 min, suggesting internalization of the molecule which increases over time (Fig. 6). Notably, no signal was remaining on the cell membrane after 18 h (Fig. 6 *D*–*G*). The colocalization with the lysosome dye indicates transport of CaRA_EGFR_ to the lysosomes. Interestingly, in comparison with the nonconditionally binding Affibody molecule targeting EGFR (40), the internalization of the CaRA_EGFR_ is higher (*SI Appendix*, Fig. S10).

### The Lysosomal Delivery of CaRA_EGFR_ Enables an IC50 of 0.8 nM When Used as a Toxin Carrier.

The cytotoxic activity of CaRA_EGFR_ conjugated to mertansine (DM1) was evaluated by treating high EGFR-expressing A-431 epidermoid cancer cells (Fig. 6*H*). Two controls were included, a nontargeting Affibody molecule conjugated to the same cytotoxin in the same way (Ztaq-DM1), and the unconjugated CaRA_EGFR_ molecule. While CaRA_EGFR_-DM1 shows dose-dependent cytotoxicity resulting in an IC50 of 0.8 nM, neither of the controls affected cell viability. Moreover, when mimicking the results of exposing healthy tissues with low EGFR-expression by exposing a low EGFR-expressing cell line (MCF-7) to CaRA_EGFR_-DM1, no impact on cell viability could be observed (*SI Appendix*, Fig. S11). When conjugating the nonconditionally binding Affibody molecule targeting EGFR (36, 40) to the same toxin, DM1, it does also not show any cytotoxic effect on cells with low EGFR-expression (*SI Appendix*, Fig. S12), but when applied to the high EGFR-expressing cells the nonconditionally binding Affibody molecule notably results in a higher IC50 in comparison to CaRA_EGFR_ since it cannot be securely determined within the same concentration interval (*SI Appendix*, Fig. S12).

## Discussion

Protein–drug conjugates (PDCs) are a growing class of targeted cancer therapies, typically utilizing antibodies that rely on efficient internalization, followed by lysosomal degradation to activate the cytotoxic drug. Herein, we present an engineering approach of a protein-based targeting agent which increases the efficiency of cellular internalization and retention, independent of receptor trafficking, through a calcium-dependent endosomal receptor release. The CaRA agent is based on a small alternative scaffold protein which offers an increased tissue penetration compared to the conventionally used full-length antibodies (41).

A calcium-dependent binder, CaRA_EGFR_, targeting EGFR, selected from a combinatorial library using phage display, has been evaluated for efficient lysosomal delivery of cytotoxic payloads (Fig. 1). The binder showed strong, calcium-dependent binding to the recombinant extracellular domain of EGFR (Fig. 2) and specificity for EGFR-expressing cancer cells (Fig. 3). At physiological calcium concentrations and pH, it displayed slow dissociation, indicating strong binding to the receptor on the cell surface. However, when calcium was reduced 1,000-fold to mimic endosomal conditions, CaRA_EGFR_ rapidly dissociates from its target within 30 seconds (Fig. 2*B*). The rapid kinetics of this release suggest that the CaRA molecule would dissociate from its target within seconds upon entering the endosomal space, functioning like a fast switch, an attribute desirable for the intended application. Notably, this calcium-dependent target release was observed at pH 7.4, showing that the interaction is pH-independent. This pH-independence would be advantageous for tumor targeting, as the pH in the vicinity of cancer cells is often lower due to rapid cell growth (42, 43). This observation is further supported by the thermal stability evaluation, which showed that the stability of the CaRA_EGFR_ is influenced by the calcium concentration, but not by pH (Fig. 2*D* and *SI Appendix*, Fig. S4). The molecular basis of binding was explored by generating an experimentally validated AlphaFold 3 model of the CaRA_EGFR_:EGFR complex. This structural model can serve as a foundation for future rational optimization of the binding affinity between CaRA_EGFR_ and EGFR.

Evaluation of the interaction between CaRA_EGFR_ and live EGFR-expressing cells further confirmed specific, calcium-dependent binding (Fig. 3 and *SI Appendix*, Fig. S5). High EGFR-expressing cells showed a significant fluorescence shift in flow cytometry when incubated with site-specific fluorescently labeled CaRA_EGFR_, a binding that only occurred in the presence of calcium (*SI Appendix*, Fig. S5). No target interaction was observed with low EGFR-expressing cell lines, regardless of calcium presence (Fig. 3*E* and *SI Appendix*, Fig. S5). According to the Human Protein Atlas (37) data on gene transcript per million (nTPM), the low EGFR expression in MCF-7 and Ramos cells correlates with the signal indicative of low expression. In contrast, higher binding signals for CaRA_EGFR_ were detected in cell lines with intermediate and high EGFR expression, which correlates with their elevated nTPM levels (*SI Appendix*, Table S2) (37).

Of importance for all drug-conjugates is that the molecules have to be efficiently internalized into the cell, and for this envisioned application also released in the endosomes, to be further transported to the lysosomes. Investigation of internalization of the CaRA_EGFR_ molecule using fluorescent microscopy indicated colocalization with lysosomes as early as after 10 min, but increasingly so over time with the strongest overlap observed after 18 h (Fig. 6), which supports our hypothesis of the internalization and trafficking mechanism. The introduction of targeting antibodies relying on endosomal dissociation by leveraging the lower calcium concentration relative to the extracellular environment has been described by others as a promising strategy to clear inflammatory cytokines and even to be used as PDCs for cancer treatment with enhanced therapeutic potential (44, 45). Noteworthy, the demonstrated in vitro cytotoxicity of 0.8 nM in the A-431 cell line (Fig. 6*H*) shows the high potency of the CaRA_EGFR_-DM1 drug conjugate. These data align with the fluorescence microscopy results, which demonstrate the lysosomal degradation of CaRA_EGFR_, as DM1 exerts its effect on microtubules after being released in the lysosomes. The IC50 is partly dependent on the conjugated cytotoxin and is expected to be in the low nanomolar range for DM1 (46), however, the nontargeting control conjugated to the cytotoxin (Ztaq-DM1) had no influence on cell viability in the tested concentration range, which shows that the targeting drives the potency. Moreover, the nonconditionally binding Affibody molecule, with higher affinity, required higher dosing to exert a cytotoxic effect on the EGFR-expressing cells (*SI Appendix*, Fig. S12). This could be hypothesized to result from its high affinity (47) rendering the Affibody to remain bound to the receptor and thereby dependent on the trafficking fate of the receptor (Fig. 5). Hence, if the receptor is recycled to the cell surface it would bring the toxic high-affinity binder along, requiring EGFR to be trafficked to the lysosomes for toxin activation. Similarly, studies have shown that the therapeutic efficacy of anti-EGFR monoclonal antibodies, such as Cetuximab, is strongly correlated with the extent of lysosomal EGFR degradation (48, 49). EGFR is described to have several endocytic trafficking routes, varying from complete recycling of the receptor to complete lysosomal degradation of the receptor, depending on which ligand that induces the receptor’s internalization (24). Whether EGFR is ultimately degraded in lysosomes or recycled to the plasma membrane directly influences the duration of receptor signaling. In our analysis of EGFR surface expression in NCI-H292 cells upon various ligand treatments, we observed that the conditional binder did not alter EGFR density, whereas the nonconditionally binding molecules did (Fig. 5*C*). Receptor degradation would diminish the possibilities for a drug carrier to enter the cell until the number of EGF receptors at the cell surface have been reestablished through protein synthesis, while if the receptor is recycled, the cell is able to immediately undergo an additional round of signaling and internalization of a PDC (24). Consequently, the receptor degradation observed for anti-EGFR antibodies like Cetuximab becomes an important determinant for their therapeutic efficacy, however, with the drawback of causing receptor downregulation at the cell membrane resulting in treatment resistance (6–7, 18). Thus, decoupling the fate of the EGF receptor itself and the therapeutic molecule like demonstrated for CaRA_EGFR_ (Fig. 5) would be desirable.

This study provides a proof-of-concept for engineering small, CaRA molecules that target cancer cell receptors for efficient intracellular release upon internalization. The small size and calcium-dependent release are anticipated to enhance tissue penetration while avoiding receptor blockade or downregulation, as the targeted receptor does not undergo the same cellular fate as the PDCs. These findings highlight the successful development of a targeting agent with tailored, conditional activation, capable of delivering toxic compounds to the lysosomes of EGFR-expressing cancer cells. This approach holds significant promise for future development into a potent therapeutic.

## Materials and Methods

### Phage Display Selections Using the CaRA library.

The target protein EGFR (70.8 kDa) was produced within the Protein Production Sweden infrastructure (Stockholm, Sweden) as previously described (50). Protein purity was confirmed by SDS-PAGE and size exclusion chromatography (SEC). EGFR was biotinylated using EZ-link Sulfo-NHS-LC-Biotin (Thermo Fisher Scientific, Waltham, MA) at a 15-fold molar excess in TBS (50 mM Tris, 150 mM NaCl, pH 7.4). Excess biotin was removed with an Illustra NAP-5 desalting column (Cytiva, Uppsala, Sweden), and the degree of biotinylation was verified by streptavidin bead binding (Dynabeads™ M-280 Streptavidin, Thermo Fisher Scientific).

Phage display selections from the CaRA library (31) against EGFR were performed as described previously (35). Four rounds of selection were carried out with decreasing target concentrations (150, 100, 50, and 25 nM). Binding occurred in TBSTC (TBS, 0.05% Tween 20, 1 mM CaCl_2_, pH 7.4), and elution was achieved using 5 mM EDTA in MBST (25 mM MES, 150 mM NaCl, 0.05% Tween 20, pH 6.0). Negative selections with 100 mM EDTA were included before rounds three and four at target concentrations matching the respective positive selections.

### Protein Production and Purification.

His_6_-CaRA_EGFR_, His_6_-CaRA_EGFR_-ABD (albumin binding domain), or His_6_-CaRA_EGFR_-Cys were produced in *Escherichia coli* BL21(DE3) and purified by Immobilized-Metal Affinity Chromatography (IMAC) or on in-house produced HSA-Sepharose for further characterization. (36) The purification was verified with SDS-PAGE, LC–MS, and SEC.

### Biolayer Interferometry.

Binding kinetics to EGFR-ECD were analyzed using His_6_-CaRA_EGFR_-ABD captured on HSA-immobilized AR2G sensors (Sartorius, Göttingen, Germany) at 20 µg/mL in TBS with 1 mM CaCl_2_. Association was measured against varying EGFR-ECD concentrations in TBS (1 mM CaCl_2_, pH 7.4). To assess calcium-dependent dissociation, the target was first associated at 1 mM CaCl_2_, followed by dissociation in buffers with decreasing calcium concentrations down to 1 µM CaCl_2_.

### Surface Plasmon Resonance.

Target binding to soluble EGFR-ECD was analyzed using a Biacore T200 instrument (Cytiva). HSA was immobilized on a CM5 sensor chip via amine coupling following the manufacturer’s protocol. Measurements were performed in TBSTC running buffer at 25 °C with a flow rate of 30 µL/min. His_6_-CaRA_EGFR_-ABD [ADB035; 50 to 500 fM affinity for human albumin (51)] was injected at 10 nM and captured on the immobilized HSA, followed by injection of EGFR at five concentrations (125, 62.5, 31.25, 15.63, and 7.82 nM). The surface was regenerated with 10 mM HCl for 30 s, and binding kinetics were evaluated using the Biacore Evaluation Software with a 1:1 Langmuir model.

### Cell Lines.

A-431 (human epidermoid carcinoma), MDA-MB-468, SK-BR-3, MCF-7 (human breast adenocarcinomas), SK-OV-3 (human ovarian adenocarcinoma), NCI-H292 (human lung carcinoma), BxPC-3 (human pancreatic adenocarcinoma), and HEK293T (epithelial-like) cells were obtained from ATCC (Manassas, VA). Cells were cultured in DMEM, McCoy’s 5A, or RPMI-1640 medium (Thermo Fisher Scientific) supplemented with 10% heat-inactivated FBS (Sigma-Aldrich, St. Louis, MO) and 100 µ/mL penicillin–streptomycin (Thermo Fisher Scientific) at 37 °C in a 5% CO_2_‚ incubator. Ramos (human B lymphocyte) cells (ATCC) were maintained in RPMI-1640 with 10% heat-inactivated FBS under the same culture conditions.

### Labeling of Protein for Flow Cytometry and Fluorescent Microscopy.

The construct His_6_-CaRA_EGFR_-Cys was site-specifically labeled with Alexa Fluor™ 488 C5 Maleimide (Thermo Fisher Scientific) on the C-terminal cysteine. The protein, at a concentration of 1 mg/ml, was reduced with 15 mM TCEP for 30 min at RT. The TCEP was removed using G-25 microspin columns (Cytiva) and the dye was added at a 20x molar excess. Excess dye was removed by buffer exchange using G-25 microspin columns. The degree of labeling was determined by measuring absorbance at 280 nm and 495 nm according to the manufacturer’s instructions.

### Flow Cytometric Assay of Specific Affinity for the Native EGFR-Receptor With and Without Calcium.

Cells were harvested at subconfluency using TrypLE™ Express Enzyme (Thermo Fisher Scientific) and resuspended to 1 × 10^6^ cells/mL in either TBS (0.1% BSA, 1 mM CaCl_2_, pH 7.4) or MBS (0.1% BSA, 5 mM EDTA, pH 6.0) to assess receptor binding in the presence or absence of calcium. Samples (100 µL; 1 × 10^5^ cells) were washed twice and incubated for 1 h at room temperature with CaRA_EGFR_-Alexa488 (500 nM to 7.62 pM). After two washes, cells were resuspended in the respective buffer and analyzed by flow cytometry (CytoFLEX, Beckman Coulter; detection at 525/40 nm).

Data represent four biological replicates, with background fluorescence (buffer-only controls) subtracted prior to averaging and SD calculation.

### Flow Cytometry Assay to Epitope map CaRA_EGFR_ Against Commercially Available Cetuximab, EGF and Z_EGFR:2377_.

To investigate whether the antibody Cetuximab and CaRA_EGFR_ share an epitope, EGFR-expressing A-431 cells were cultured and trypsinated as described earlier. The cells were washed and suspended in TBS, 0.1% BSA, and 1 mM CaCl_2_ and incubated with human EGF (Sigma-Aldrich), Cetuximab, or Z_EGFR:2377_ at 100 nM for 1 h at 4 °C first, following one wash, binding with 100 nM Alexa488-labeled CaRA_EGFR_ was evaluated by subsequent incubation for 30 min on ice. After another wash, the median fluorescent intensity of triplicate samples was collected by flow cytometry using a CytoFLEX instrument as described above. A background signal of unstained cells was subtracted.

### Flow Cytometry Assay to Determine the Level of Cell Surface-Expressed EGFR Upon Incubation With Various Ligands.

To assess whether natural ligands (EGF, BTC) or engineered anti-EGFR binders (Cetuximab, Z_EGFR:2377_, CaRA_EGFR_) affect EGFR surface expression, NCI-H292 cells were seeded at 3 × 10^5^ cells/well in 6-well plates. After attachment, cells were treated with 100 nM of each ligand for 15 min to 24 h. Following incubation, cells were washed, detached, and resuspended in PBS with 0.1% BSA, then stained with anti-EGFR antibody (clone 199.12; 2 µg/mL) for 30 min on ice in the dark, followed by Alexa Fluor 647–conjugated goat anti-mouse secondary antibody (Thermo Fisher A-21235). After washing, median fluorescence intensity was measured by flow cytometry (CytoFLEX, Beckman Coulter). Surface expression levels are presented as the percentage of untreated control cells.

### Circular Dichroism Spectroscopy (CD).

The structural stability of CaRA_EGFR_ was analyzed by circular dichroism (CD) spectroscopy using a Chirascan spectrometer (Applied Photophysics, UK). Measurements were performed with a 1 mm path length and a protein concentration of 0.4 mg/mL. The secondary structure was recorded from 260 to 195 nm at 20 °C, followed by variable temperature measurements (VTM) at 221 nm during a temperature ramp of 5 °C/min from 4 °C to 95 °C. Fractional unfolding was calculated as described previously (24). Samples were prepared in MBS (pH 6.0 or 6.5) or TBS (pH 7.4) supplemented with 1 mM, 500 µM, 250 µM, 50 µM, or 1 µM CaCl_2_ to assess pH- and calcium-dependent structural changes.

### Fluorescence Microscopy.

Subconfluent A431 cells were harvested by trypsinization and seeded in 96-well plates at 5,000 cells per well, followed by overnight incubation at 37 °C and 5% CO_2_. The next day, cells were treated with 2 µM CaRA_EGFR_–Alexa 488 and 50 nM LysoTracker™ Deep Red (Thermo Fisher Scientific) in complete medium for 10 min at room temperature, washed once, and imaged using a Leica SP8 confocal microscope with a 40× dry objective and 488 nm/638 nm excitation. Cells were then incubated for 30 min at 37 °C, 5% CO_2_‚ to allow internalization, and Hoechst nuclear stain was added 10 min prior to imaging. Additional images were acquired after 30 min, 2 h, and 18 h using 405 nm excitation. Image processing was performed in Fiji for background subtraction and in Python (OpenCV, SciPy, NumPy, scikit-image) for analysis. Otsu segmentation was applied to identify fluorescence masks, and colocalization with lysosomes was quantified as percentage overlap at each time point.

Moreover, the harvested subconfluent A431 cells were seeded in ibidi µ-Slide 8 Well Glass Bottom chamber slides at 1 × 10^5^ cells/well and incubated overnight at 37 °C, 5% CO_2_. The following morning, the medium was replaced with fresh medium containing 50 nM LysoTracker™ Blue DND-22 (Thermo Fisher Scientific) 30 min prior to imaging. Live-cell imaging was performed using a Zeiss 980 Airyscan2 confocal microscope with a 40×/1.2 NA water immersion objective at 373 nm and 493 nm excitation, in an incubated chamber set to 37 °C, 5% CO_2_. After a 10 s baseline recording, 2 µM CaRA_EGFR_–Alexa488 or Z_EGFR:2377_–Alexa488 was added directly to the imaging chamber. Recordings were made in three independent replicates, with additional imaging at 60 min and 120 min to monitor internalization.

HEK293T cells (3 × 10^5^) were seeded in ibidi µ-Slide 8 Well Glass Bottom chamber slides and transfected after 24 h with 3 µg EGFR-FR plasmid (Addgene #179263, gift from Jared Toettcher) using PEI MAX (VWR) or left untransfected (52). Live-cell imaging was performed on a Zeiss 980 Airyscan2 confocal microscope with a 40×/1.2 NA water immersion objective at 405 nm and 488 nm excitation, in an incubated chamber set to 37 °C, 5% CO_2_. After a 10 s baseline recording, 2 µM CaRA_EGFR_–Alexa488 was added directly to the imaging chamber. Imaging was performed in three independent replicates using interleaved 488 and 561 nm excitation in fast Airyscan SR-4Y mode at 15 z-positions spaced 0.21 µm (volume acquisition: 2.1 s). Average full-frame intensities were measured from maximum intensity projections and plotted over time. Consecutive recordings were taken 24 h posttransfection using the same procedure.

Image processing and analysis was made using the colocalization module in Zen 3.10 (Carl Zeiss). Quantification of fluorescence intensity (Fig. 6) was made in Fiji (https://imagej.net).

### Immunofluorescence Staining and Microscopy.

A-431 cells were harvested and seeded in chamber slides at a concentration of 100 000 cells/well. The slides were incubated overnight at 37 °C and 5% CO_2_. The next morning, the cell media were aspirated and either CaRA_EGFR_-Alexa488 or Z_EGFR:2377_-Alexa488 was supplied to the cells at a concentration of 100 nM diluted in complete media. The slides were incubated at 37 °C and 5% CO_2_ during 15 min respectively 60 min to allow for internalization of the construct. After the specified time, cells were washed twice with PBS and fixed in 4% paraformaldehyde. Subsequent to fixation, cells were permeabilized and blocked in PBS containing 1% BSA. Immunofluorescence labeling of EGFR was performed using a mouse monoclonal primary antibody (anti-EGFR IgG_2A_, clone 199.12 Thermo Fisher Scientific) and an isotype specific secondary antibody (goat anti-mouse IgG) conjugated to Alexa647 (Invitrogen). The cell nuclei were stained with DAPI supplemented in the mounting media prior to imaging using a Zeiss980 Airyscan2 confocal microscope.

### Conjugation to Cytotoxin DM1.

His_6_-CaRA_EGFR_-Cys as well as a negative control, Affibody molecule His_6_-Z_taq_-Cys (53), were produced and purified as described above for conjugation to the cytotoxin mertansine (DM1) through thiol-coupling. After reduction with 20 mM DTT for 30 min at 37 °C, the protein was buffer exchanged to 200 mM NH_4_Ac pH 6.5 using a NAP5 column (Cytiva). A 2x molar excess of mc-DM1 (CD Chemicals) was added and incubated overnight at room temperature. The conjugated product was separated from nonconjugated through reverse-phase HPLC with a gradient of H_2_O/acetonitrile with 0.1% TFA using a gradient of 20 to 80% at 40 °C. Distinct peaks were collected and analyzed using MALDI TOF/TOF, pooling peaks with the correct molecular weight of the conjugated product. The conjugated protein was freeze-dried and resuspended in a low volume of TBS and the concentration was determined through measuring the absorbance at 280 nm.

### Cytotoxicity Assay.

The human cell line A-431 (epidermoid carcinoma) was cultured as previously described, seeded in a 96-well plate at a density of 5000 cells/well in 100 μl and incubated at 37 °C, 5% CO_2_ overnight allowing the cells to adhere prior to addition of the different constructs, in a concentration series starting at 100 nM with each concentration in triplicates. The cells were incubated for 48 h at 37 °C and 5% CO_2_ before the viability was measured using the cell counting kit CCK8 (Sigma). The absorbance at 450 nm was measured using a ClarioStar plate reader. The cell viability was normalized to untreated cells using Prism 9 (GraphPad Software, LLC.), the data were fitted to a sigmoid function and IC50 values were calculated from three independent experiments.

### Cryo-EM Sample Preparation and Data Collection.

Equimolar ratios of CaRA_EGFR_ and the extracellular domain of EGFR at 25 μM were incubated in TBS (50 mM Tris; 150 mM NaCl pH 7.4) with 1 mM CaCl_2_ for 1 h at room temperature. A Quantifoil R 2/2 Cu 300 grid (Quantifoil Micro Tools, Prod. No. 658-300-CU) was glow-discharged for 30 s at 50 mA before the addition of a 4 μL CaRA_EGFR_:EGFR sample. Grids were then flash-frozen in liquid ethane using a FEI Vitrobot (Thermo Fisher Scientific) and subsequently blotted at 100% humidity, 4 °C, and a blotting time of 5 s. Micrographs were collected at the Umeå Core Facility for Electron Microscopy on a 200Â kV Glacios system (Thermo Fisher Scientific) equipped with a Falcon 4i direct electron detector. EPU (Thermo Fisher Scientific) was used for the automated data collection of a total of 3001 movies with 40 frames at a total dose of 50 e^−^/Å^2^ and a pixel size of 0.953 Å.

### Cryo-EM Image Processing and Structural Visualization.

The dataset was processed using cryoSPARC (v4.6.0-4.6.2) according to the processing scheme displayed in *SI Appendix*, Fig. S13. (54) Micrographs were manually curated, resulting in a final dataset of 1642 exposures. Particles were autopicked using a Gaussian blob picker and subsequently classified in 2D. Promising classes were used for an ab-initio reconstruction and subsequent refinement. After curation, an initial volume was used to generate templates for a template-picker. Template-based particles were then classified in 2D and 3D and subjected to ab-initio modeling, followed by heterogenous, homogenous, and finally nonuniform refinement (55).

The particles of the most promising volume were then used for reference-based motion correction and again classified in 2D to further remove suboptimal particles. The final stack of 161 482 particles was used for ab-initio modeling and nonuniformly refined without symmetry constraints. While cryoSPARC states a resolution of 3.89 Å, the lack of distinguishable features of secondary structures that should be visible at such levels suggests a resolution of 6 to 8 Å. The structure of CaRA_EGFR_ in complex with the soluble domain of EGFR in presence of Ca^2+^ was predicted using AlphaFold 3 using the AlphaFold Server. (38) The AlphaFold 3 model was docked into the EM density map using Phenix (v1.21.2) (56) and the results were visualized by superposition in USCF ChimeraX (v1.9). (57).

## Supplementary Material

Appendix 01 (PDF)

## Data Availability

All study data are included in the article and/or *SI Appendix*.
