# Supplementary material for: Engineered calcium-regulated affinity protein for efficient internalization and lysosomal toxin delivery
Source: Proc Natl Acad Sci U S A. 2025 Nov 25;122(48):e2509081122. doi: 10.1073/pnas.2509081122 (PMC12685030; doi:10.1073/pnas.2509081122)
Supplement: Supplementary file 1 — Appendix 01 (PDF) [file pnas.2509081122.sapp.pdf]

# Supplementary material

## Engineered calcium-regulated affinity protein for efficient internalization and lysosomal toxin delivery

Malin Jönsson[1*], Marit Möller[1*], Leon Schierholz[4], Nicolai Dorka[2], Hanna Tegel[1], Emma Lundberg[2], Mathias Uhlén[1], Magnus Wolf-Watz[5], Hjalmar Brismar[3], Sophia Hober[1#]

[1]Department of Protein Science, SciLifeLab, KTH-Royal Institute of Technology, Stockholm, Sweden

[2]Department of Bioengineering, Stanford University, Stanford, CA-4525, USA

[3]Department of Applied Physics, SciLifeLab, KTH-Royal Institute of Technology, Stockholm, Sweden

[4]Department of Molecular Biology, Umeå University, SE-901 87, Umeå, Sweden

[5]Department of Chemistry, Umeå University, SE-901 87, Umeå, Sweden

*Co-first authors with equal contribution

[#]Corresponding author, sophia@kth.se, phone +46 8 790 87 94, fax +46-5537-8481

**Table S1.** Calculated melting temperatures of variable temperature measurements between 4 °C and 95 °C of all characterized pH conditions in the presence of 1 mM - 1 µM calcium resulted in the following calculated melting temperatures.

| Calcium concentration | *pH 7.4* | *pH 6.5* | *pH 6.0* |
|---|---|---|---|
| ***1 mM*** | *45.40* | *46.75* | *46.14* |
| ***500 µM*** | *41.50* | *43.17* | *43.92* |
| ***250 µM*** | *39.86* | *41.45* | *41.62* |
| ***50 µM*** | *37.77* | *37.94* | *38.90* |
| ***1 µM*** | *36.45* | *36.32* | *37.48* |

**Table S2.** Number of EGFR transcripts per million (nTPM) on the cell lines used for flow cytometric analysis, confocal microscopy, and *in vitro* cytotoxicity studies according to the Human Protein Atlas (www.proteinatlas.org). The experiments included the following cell lines; skin cancer derived A-431 cells (high expression), breast cancer derived MDA-MB-468 and SK-BR3 cells (intermediate expression), ovarian cancer derived SK-OV-3 cells (intermediate expression), lung cancer derived NCI-H292 cells (intermediate expression), pancreatic cancer derived BxPC-3 cells (intermediate expression), and two cell lines with no or low detected EGFR-expression; Ramos cells (lymphoma) and MCF-7 cells (breast cancer). [36, 37] Moreover, the reported mean, minimum and maximum nTPM values for respective cancer tissue (skin, breast, pancreatic, lung and lymphoma) that the cell lines are representing is included and the number of patients these values were derived from. [36, 37]

| **Cell lines** | ***EGFR*** | ***HER2*** | ***HER3*** | ***Type of cancer*** | ***Cancer tissue mean*** | ***Cancer tissue min*** | ***Cancer tissue max*** | ***Number of patients*** |
|---|---|---|---|---|---|---|---|---|
| | *nTPM* | *nTPM* | *nTPM* | | *nTPM* | *nTPM* | *nTPM* | |
| **A-431** | *2978* | *58.3* | *42.1* | *Skin cancer* | *ND* | *ND* | *ND* | *0* |
| **MDA-MB-468** | *702.8* | *17.6* | *23.0* | *Breast cancer* | *10* | *0* | *1829* | *1022* |
| **BxPC-3** | *98.5* | *43.0* | *35.4* | *Pancreatic cancer* | *13* | *0* | *85* | *176* |
| **NCI-H292** | *86.7* | *62.0* | *22.6* | *Lung cancer* | *58* | *0* | *1060* | *489* |
| **SK-BR-3** | *37.2* | *2448* | *91.0* | *Breast cancer* | *10* | *0* | *1829* | *1022* |
| **SK-OV-3** | *35.6* | *2047.9* | *1.0* | *Breast cancer* | *10* | *0* | *1829* | *1022* |
| **MFC-7** | *1.4* | *47.1* | *60.1* | *Breast cancer* | *10* | *0* | *1829* | *1022* |
| **Ramos** | *0.0* | *1.4* | *0.1* | *Lymphoma* | *ND* | *ND* | *ND* | *0* |

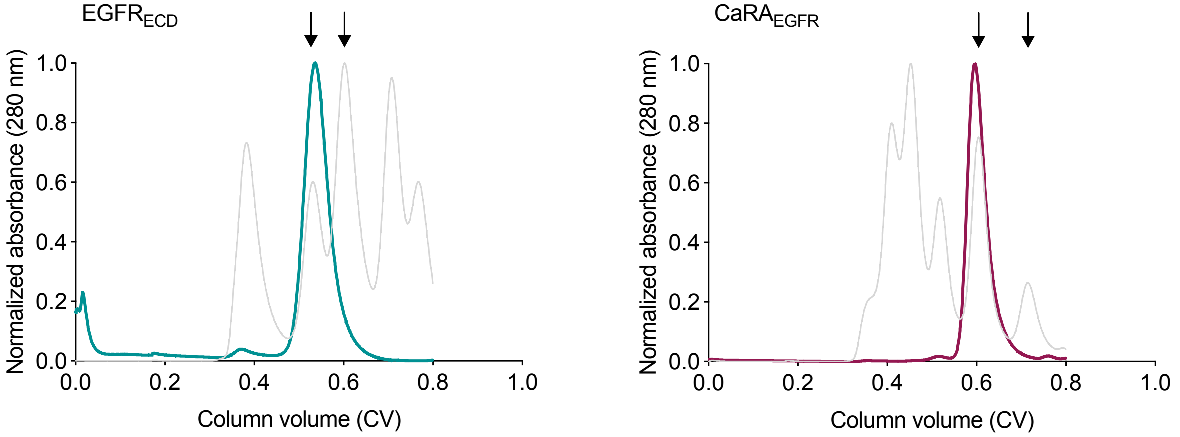


**Figure S1.** SEC chromatograms showing the target selected against; the extracellular domain of EGFR (teal) and the characterized variant CaRA$_{EGFR}$ (burgundy). The expected molecular weight was confirmed for both, approximately 71 kDa for EGFR and approximately 8.5 kDa of the calcium-regulated variant, in monomeric form. Indicated by arrows is the molecular size of two reference proteins (158 kDa and 44 kDa for EGFR$_{ECD}$, respective 13.7 kDa and 6.50 kDa for CaRA$_{EGFR}$) within each calibrant (grey) for comparison. Both proteins are known to run higher than their expected size from reducing conditions on SDS-PAGE.

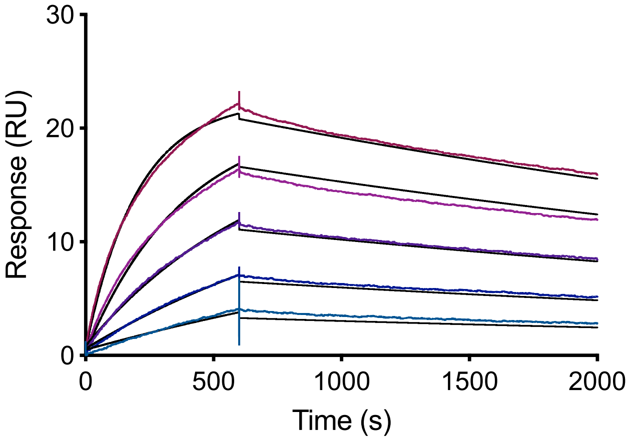


**Figure S2**. SPR sensorgram showing the interaction of EGFR-ECD injected over CaRA$_{EGFR}$-ABD (ABD035 with a reported 50-500 fM apparent affinity for human serum albumin [51]) captured on immobilized human serum albumin (HSA) at concentrations of 125 nM, 62.5 nM, 31.25 nM, 15.63 nM and 7.82 nM. Black curves represent the fits using a 1:1 binding interaction model, resulting in the following kinetic constants: $k_a = 3.82*10^4$ $M^{-1}$ $s^{-1}$ , $k_d = 2.08*10^{-4}$ and $K_D = 5.45*10^{-9}$ M.

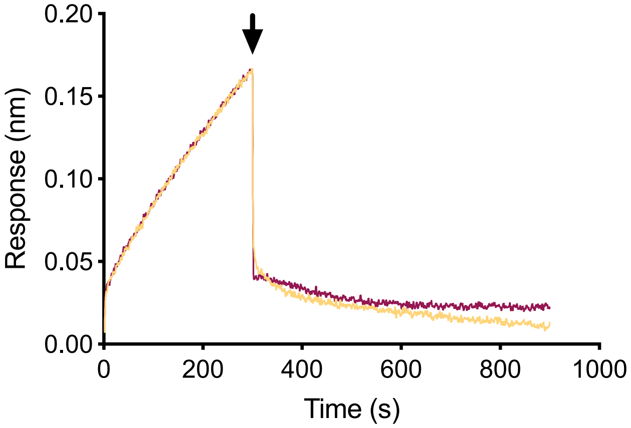


**Figure S3**. BLI sensorgram showing the association of EGFR-ECD to CaRA$_{EGFR}$ captured on the His-tag in buffer at pH 7.4 supplemented with 1 mM $CaCl_2$ and dissociation in buffer at pH 7.4 (burgundy) or pH 5 (yellow) supplemented with 2 µM $CaCl_2$. The arrow indicates the start of the dissociation in the different buffers and shows a similar release independent of the lower pH.

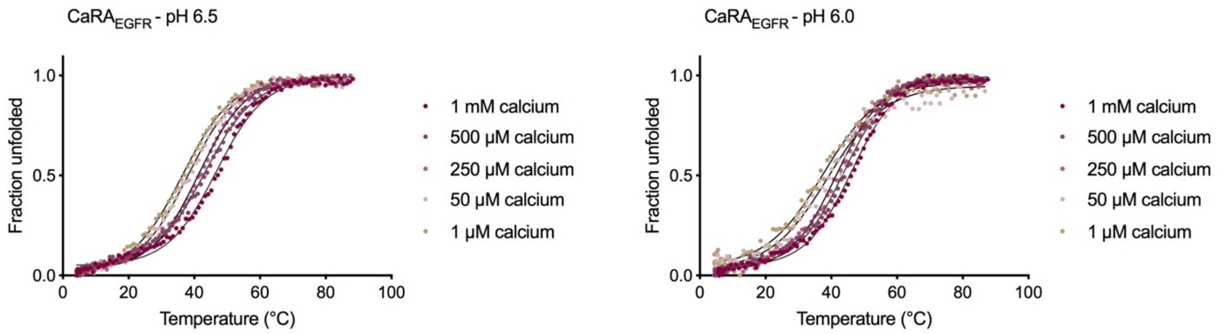


**Figure S4.** Variable temperature measurements between 4 °C and 95 °C of the characterized pH conditions (6.0 and 6.5) in the presence of 1 mM - 1 µM calcium.

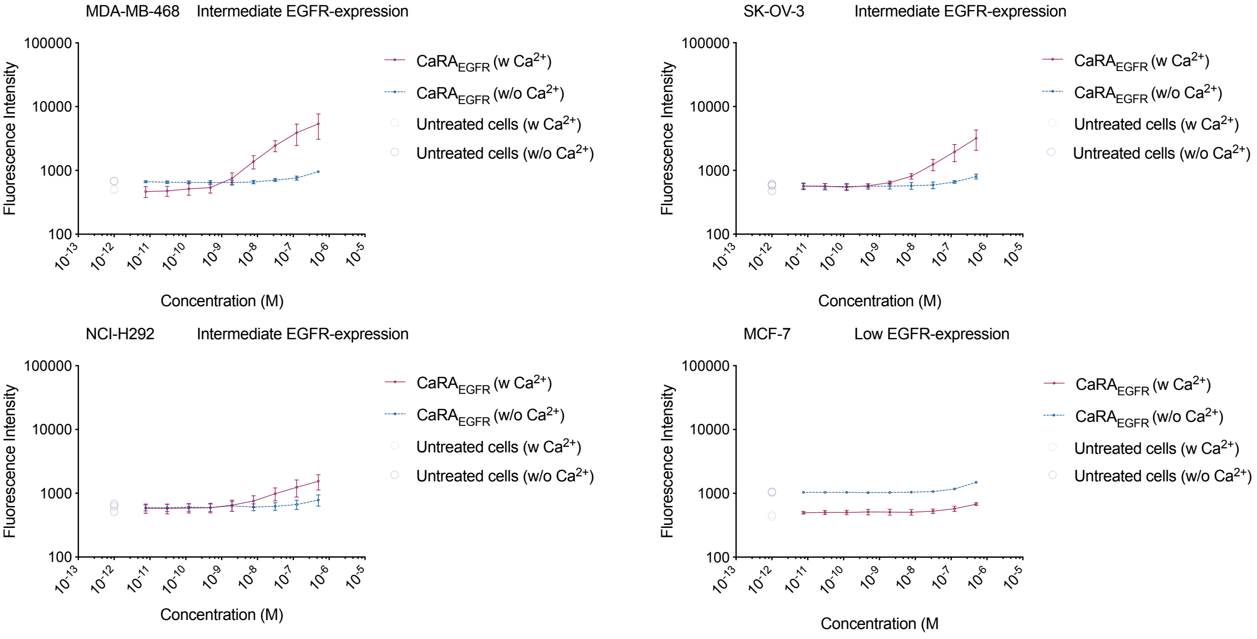


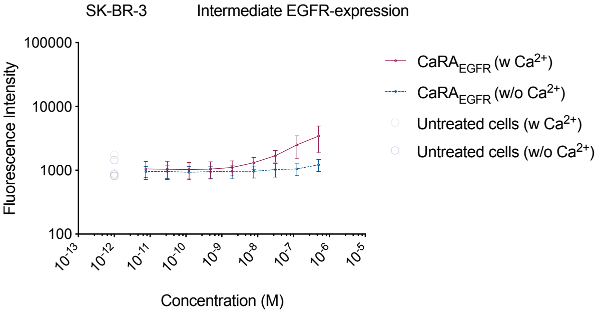


**Figure S5.** Flow cytometric data of a concentration series ranging from 500 nM to 7.62 pM CaRA$_{EGFR}$-Alexa488 incubated with various EGFR-expressing cancer cells in the presence (burgundy) or absence (blue) of calcium. For the i ntermediate EGFR-expressing cell lines a concentration dependent shift in fluorescent signal can be observed when CaRA$_{EGFR}$ is supplemented with calcium while in a calcium-free setting the signal overlaps with blank cells (grey circles).
The shift in fluorescence signal in the presence nor absence of calcium cannot be observed for MCF-7 which is expected since it according to literature does not express the EGF receptor (Table S2).

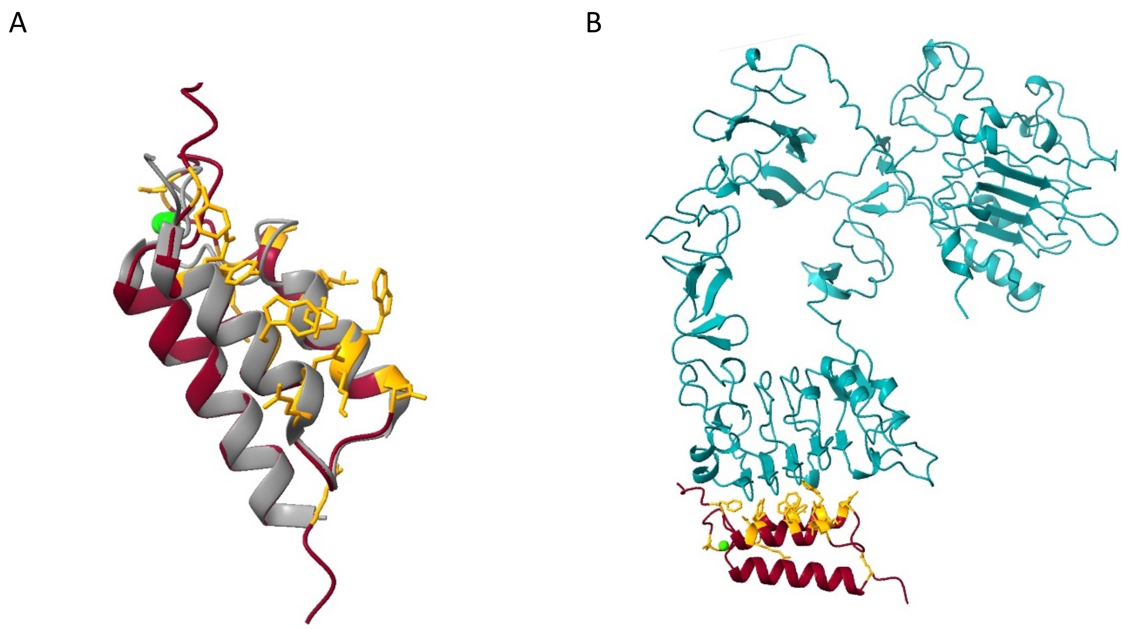


**Figure S6. Alphafold3 predicted EGFR binding surface on CaRA$_{EGFR}$**. A) Superimposition of the CaRA$_{EGFR}$ Alphafold3 model (burgundy) on the parental Z$_{Ca}$ structure (gray) (chain E in 6FGO.pdb). The amino acid residues that have been introduced through the selection towards EGFR are colored in yellow on CaRA$_{EGFR}$. B) Alphafold3 model of the CaRA$_{EGFR}$:EGFR complex. CaRA$_{EGFR}$ is shown in burgundy while EGFR is shown in blue. The amino acid residues that have been introduced through the selection towards EGFR are colored in yellow on CaRA$_{EGFR}$. Calcium ions are shown as green spheres in both panels.

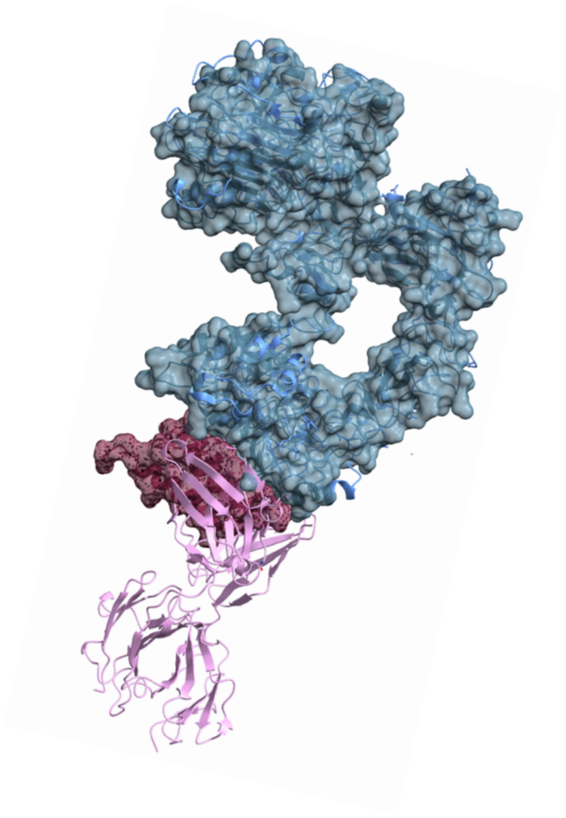

**Figure S7.** A crystal structure (PDB: 1YY9, [39]) depicting the interaction between human EGFR and cetuximab. On this structure, the proposed complex between EGFR and CaRA$_{EGFR}$ has been superimposed, further supporting the results from the epitope binning experiments (Figure 4C).

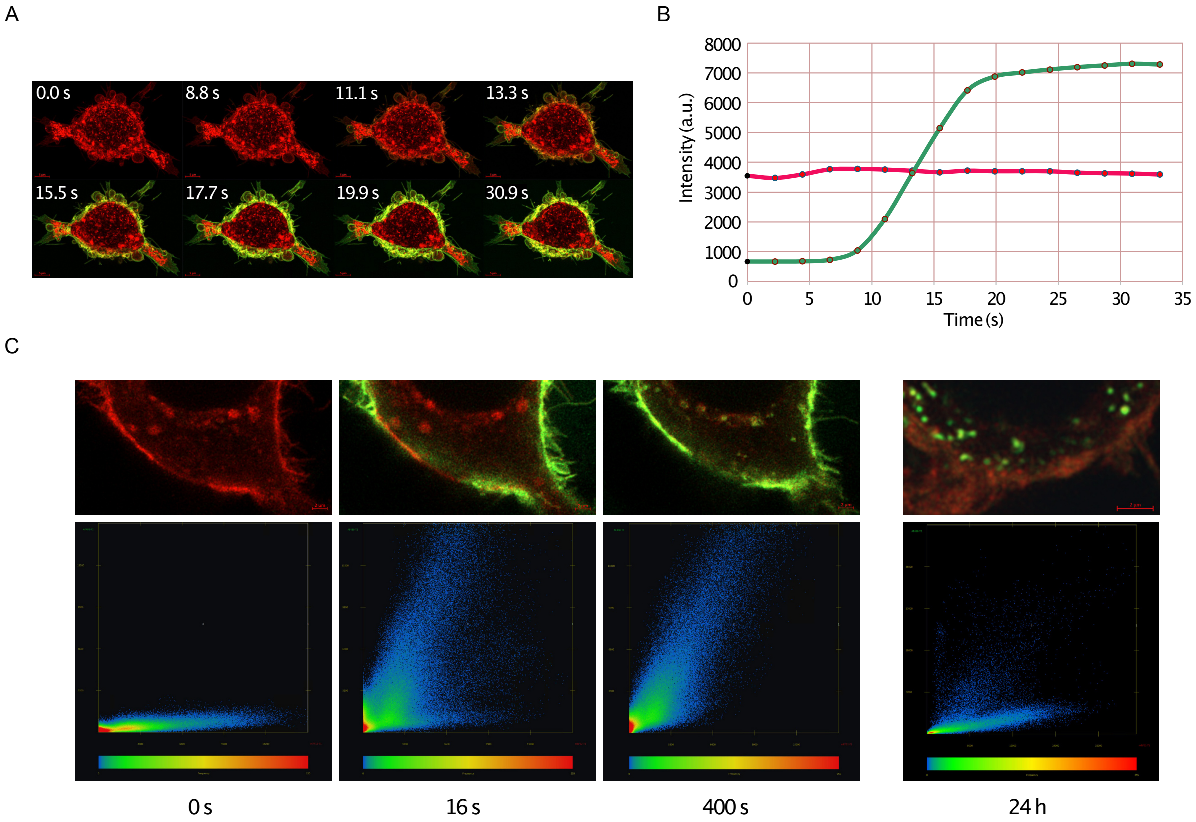


**Figure S8. Dynamic detachment of internalized CaRA$_{EGFR}$ from EGFR-FR in transfected HEK293T cells.** A) The Alexa488 fluorescently labelled CaRA$_{EGFR}$ (green) was added to live HEK293T transfected cells expressing EGFR-FR (red) and within 10 s upon addition to the cells the surface exposed receptors were saturated and internalisation of CaRA$_{EGFR}$ could be visualized. B) Time course of CaRA$_{EGFR}$ binding to EGFR shows a rapid binding and accumulation occuring within 10 seconds after addition of fluorescently labelled CaRA$_{EGFR}$ to the cell medium. C) Colocalization analysis of CaRA$_{EGFR}$ and EGFR. Upper row shows a zoom in of a cell expressing EGFR, at t = 0 s labelled CaRA$_{EGFR}$ is added to the cell medium and accumulation followed by internalization of CaRA$_{EGFR}$ is observed. In the lower row, 2D histograms of red EGFR signal on x-axis and green CaRA$_{EGFR}$ signal on y-axis is shown. The colocalization histogram show how CaRA$_{EGFR}$ colocalize with EGFR initially but at 24 h, a discrete localization of CaRA$_{EGFR}$ without EGFR is present, also visible as solid green vesicle-like structures in the image at 24 h.

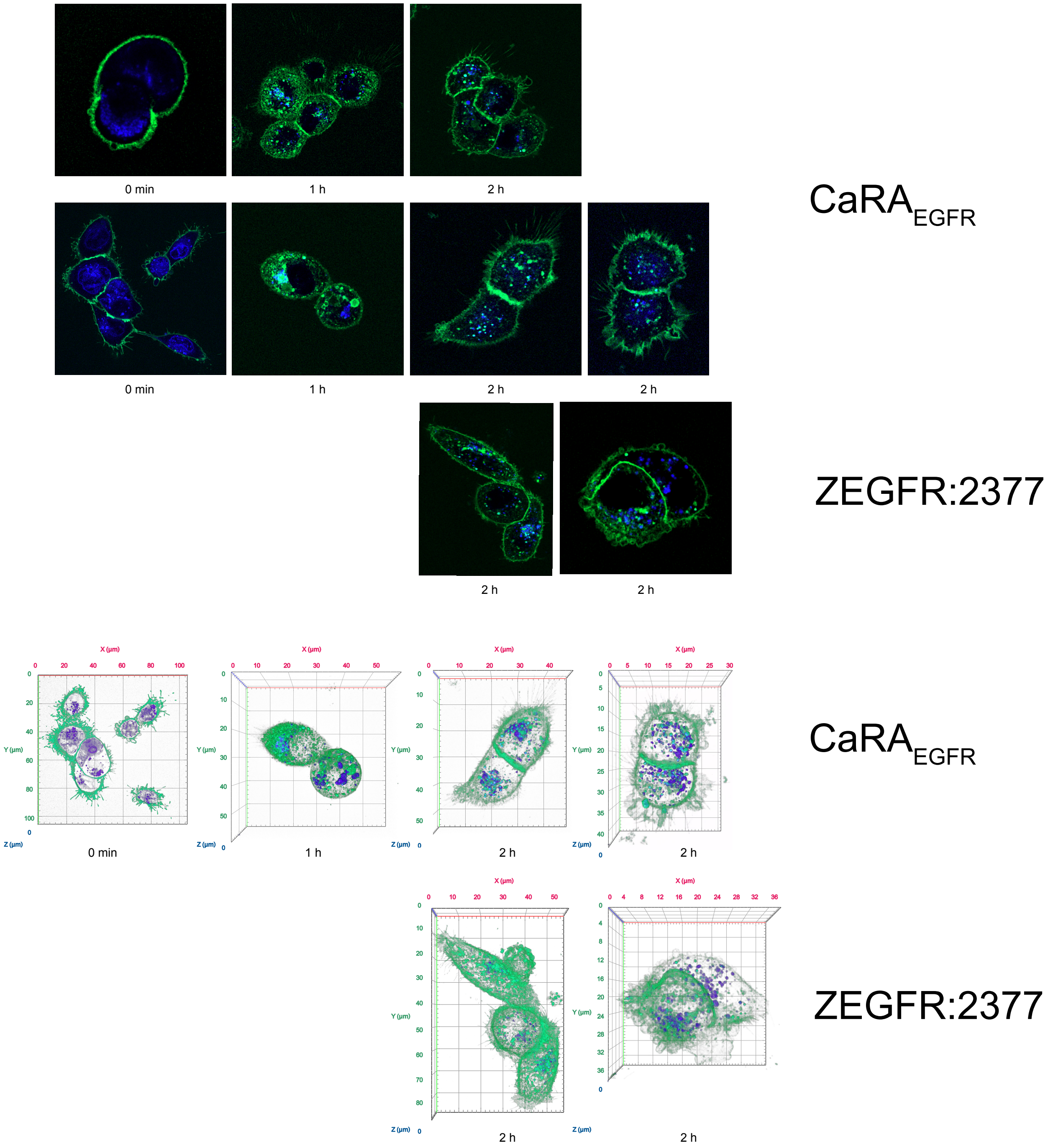


**Figure S9. Single z-stack 2D images and corresponding z-stack 3D images of CaRA$_{EGFR}$-Alexa488 and a non-conditionally binding Affibody molecule, ZEGFR:2377-Alexa488, shows that CaRA$_{EGFR}$-Alexa488 is colocalizing with the dark blue lysosome dye during the time lapse of 2h.** The Alexa488 fluorescently labelled CaRA$_{EGFR}$ (green) was incubated with A-431 cells for 0 min, 1 h and 2 h at 37°C together with lysosome dye (blue). The colocalization of CaRA$_{EGFR}$ with the lysosomes appear as turquoise in the 2D (single z-plane images) and in the corresponding z-stacked 3D maps. The non-conditionally binding Affibody molecule ZEGFR:2377-Alexa488 (green) was also incubated with A-431 cells for 2 h at 37°C together with lysosome dye (blue), however it appears to be mostly located at the cell membrane still after the indicated time period.

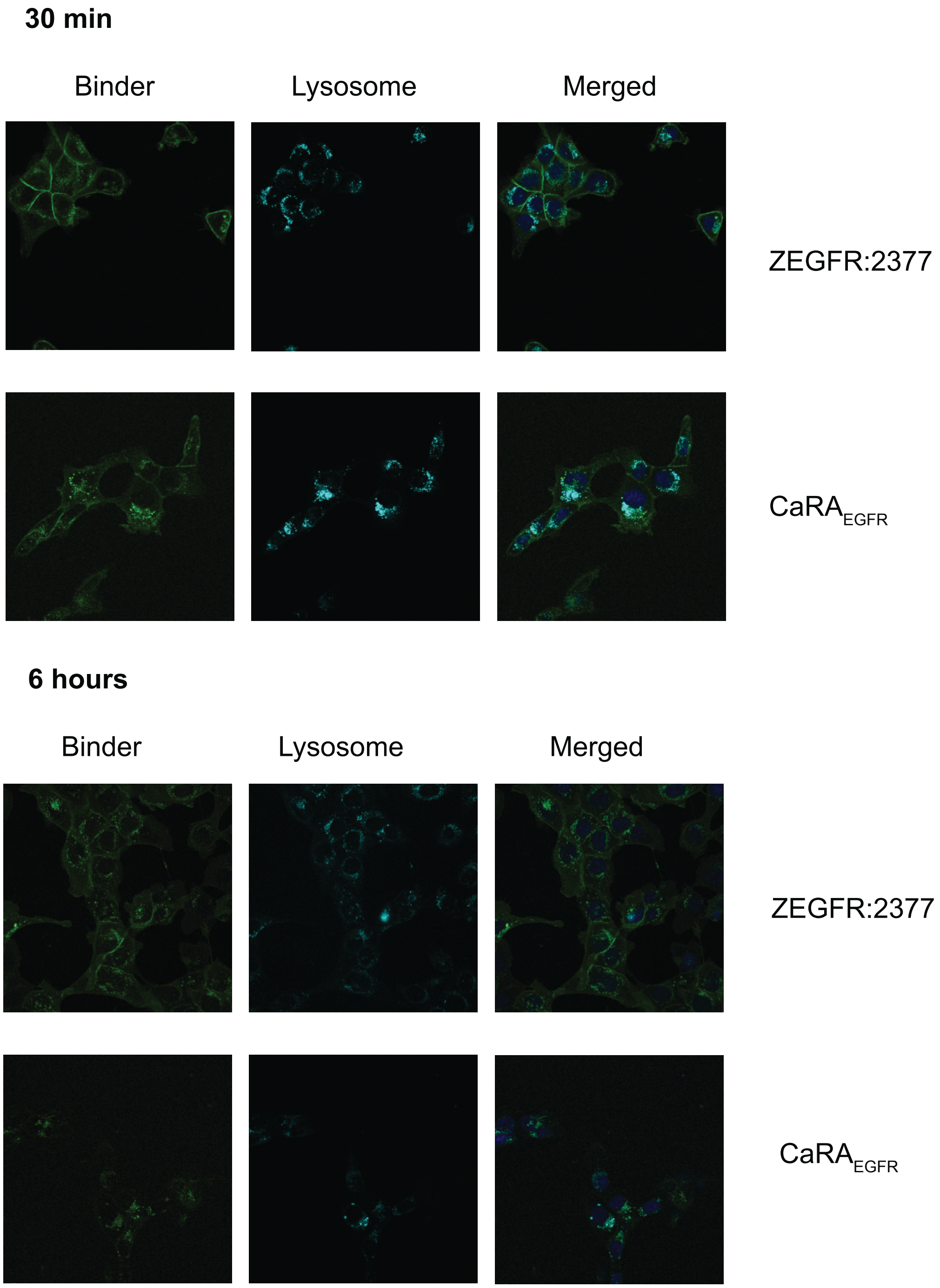


**Figure S10.** Confocal microscopy images of EGFR-expressing A-431 cells exposed to CaRA$_{EGFR}$ or a non-conditional high affinity targeting Affibody molecule (ZEGFR:2377). The Alexa488 fluorescently labelled binders (green) CaRA$_{EGFR}$ and ZEGFR:2377 was separately incubated with high EGFR-expressing cells at 37 °C to allow for internalization of the receptor during 30 min and 6 h together with lysosome dye (cyan). Cell nuclei stain (Hoechst) is shown in blue. It can be seen that both binders are localized to the membrane were EGFR is commonly expressed after 30 min. However, the confocal micrographs from the centre of the cells captured after 6 h at 40x magnification at excitation 405 nm, 488 nm, and 638 nm displays that CaRA$_{EGFR}$ has been transported to the lysosomes and colocalizes with the signals from the lysosome dye (cyan) closer to the cell nuclei (blue), while ZEGFR:2377 is still mostly visible on the membrane.

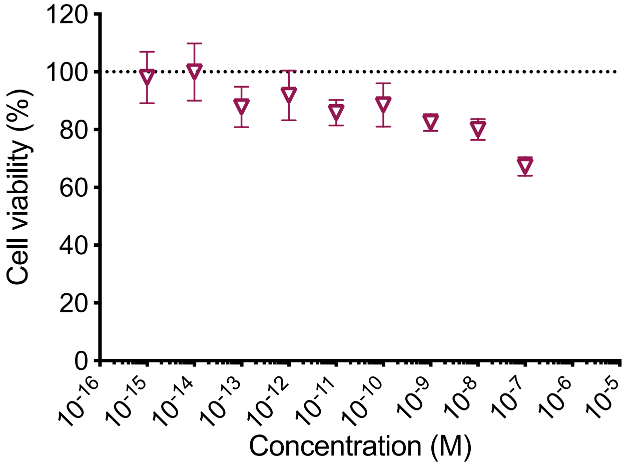


**Figure S11.** Cytotoxic assay result of low EGFR-expressing MCF-7 cells exposed to $CaRA_{EGFR}$ shows, as expected, low impact on cell viability, highlighting the specificity of $CaRA_{EGFR}$

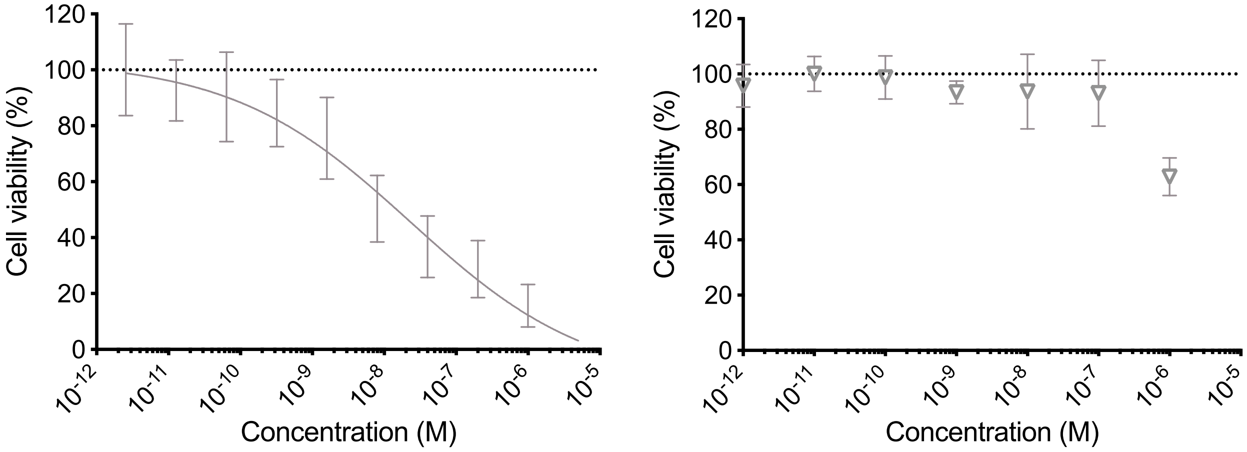


**Figure S12.** Cytotoxic assay results of high (A-431) and low EGFR-expressing (MCF-7) cells exposed to a non-conditional high affinity targeting Affibody molecule (ZEGFR:2377) shows a cell killing effect on A-431 (left) but not on MCF-7 (right). However, no $IC_{50}$ could be calculated for A-431 within this concentration interval.

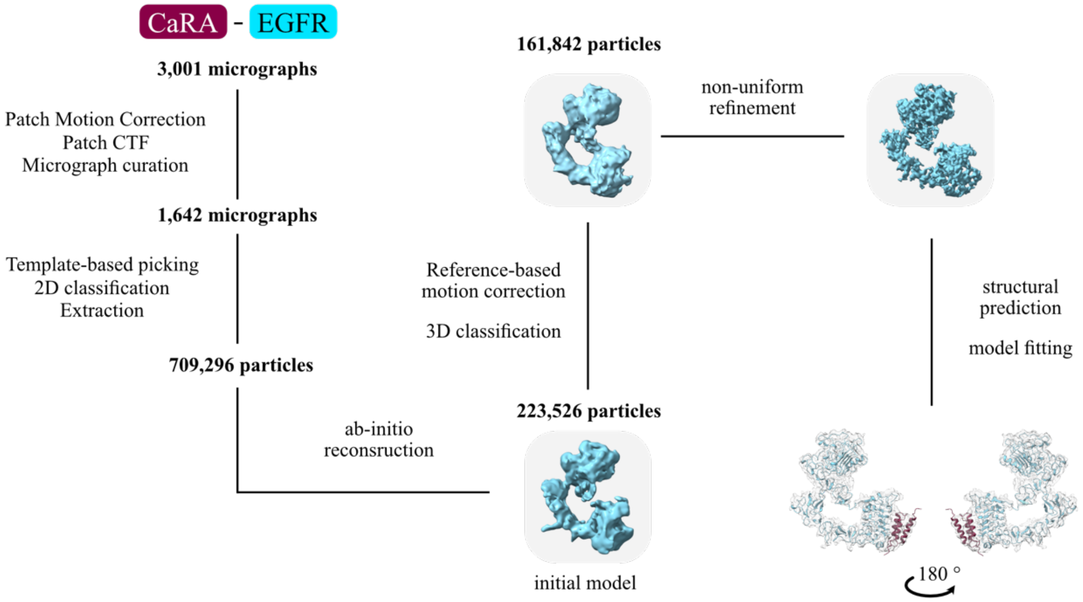


**Figure S13. Cryo-EM data processing scheme.** Outline of the workflow performed in cryoSPARC to process the data collected of the associated $CaRA_{EGFR}$:EGFR complex. The resulting cryo-EM density map was used to identify the binder's location in the complex together with a structural model predicted with AlphaFold3. [38]
